# Holistic view of the seascape dynamics and environment impact on macro-scale genetic connectivity of marine plankton populations

**DOI:** 10.1186/s12862-023-02160-8

**Published:** 2023-09-01

**Authors:** Romuald Laso-Jadart, Michael O’Malley, Adam M. Sykulski, Christophe Ambroise, Mohammed-Amin Madoui

**Affiliations:** 1 Génomique Métabolique, Genoscope, Institut François Jacob, CEA, CNRS, Univ Evry, Université Paris-Saclay, Evry, France; 2 Research Federation for the Study of Global Ocean Systems Ecology and Evolution, FR2022/Tara Oceans GO-SEE, 3 rue Michel-Ange, Paris, France; 3 STOR-i Centre for Doctoral Training/Department of Mathematics and Statistics, Lancaster University, Lancaster, UK; 4 LaMME, CNRS, Univ Evry, Université Paris-Saclay, Evry, France; 5 Service d’Etude des Prions et des Infections Atypiques (SEPIA), Institut François Jacob, Commissariat à l’Energie Atomique et aux Energies Alternatives (CEA), Université Paris Saclay, Fontenay-Aux-Roses, France; 6 Équipe Écologie Évolutive, UMR CNRS 6282 BioGéoSciences, Université de Bourgogne Franche-Comté, 21000 Dijon, France

**Keywords:** Population genetics, Marine plankton, Isolation-by-currents, Metagenomics

## Abstract

**Background:**

Plankton seascape genomics studies have revealed different trends from large-scale weak differentiation to microscale structures. Previous studies have underlined the influence of the environment and seascape on species differentiation and adaptation. However, these studies have generally focused on a few single species, sparse molecular markers, or local scales. Here, we investigated the genomic differentiation of plankton at the macro-scale in a holistic approach using *Tara* Oceans metagenomic data together with a reference-free computational method.

**Results:**

We reconstructed the *F*_ST_-based genomic differentiation of 113 marine planktonic taxa occurring in the North and South Atlantic Oceans, Southern Ocean, and Mediterranean Sea. These taxa belong to various taxonomic clades spanning Metazoa, Chromista, Chlorophyta, Bacteria, and viruses. Globally, population genetic connectivity was significantly higher within oceanic basins and lower in bacteria and unicellular eukaryotes than in zooplankton. Using mixed linear models, we tested six abiotic factors influencing connectivity, including Lagrangian travel time, as proxies of oceanic current effects. We found that oceanic currents were the main population genetic connectivity drivers, together with temperature and salinity. Finally, we classified the 113 taxa into parameter-driven groups and showed that plankton taxa belonging to the same taxonomic rank such as phylum, class or order presented genomic differentiation driven by different environmental factors.

**Conclusion:**

Our results validate the isolation-by-current hypothesis for a non-negligible proportion of taxa and highlight the role of other physicochemical parameters in large-scale plankton genetic connectivity. The reference-free approach used in this study offers a new systematic framework to analyse the population genomics of non-model and undocumented marine organisms from a large-scale and holistic point of view.

**Supplementary Information:**

The online version contains supplementary material available at 10.1186/s12862-023-02160-8.

## Introduction

Marine species from epipelagic plankton are drifting organisms that are abundant in the global ocean, play an active role in Earth’s biogeochemical cycles, and form a complex trophic web with high taxonomic diversity based on fish resources [1–9]⁠. Understanding the present connectivity between populations or communities of plankton is thus crucial to apprehend upheavals due to climate change in oceans [10, 11]. Due to their potentially high dispersal and large population size, planktonic species have long been thought to be homogenous and highly connected across oceans, but this assumption has been challenged by empirical studies over the past two decades [12]⁠. Planktonic species are characterized by theoretically high population effective sizes [13, 14], which reduces the power of genetic drift and makes selection and beneficial mutations stronger drivers of their evolution, as exemplified in the SAR11 Alphaproteobacteria [15]⁠, but the balance between neutral evolution and selection is still debated [16, 17]. Furthermore, plankton evolution also seems to be strengthened by acclimation through variations in gene expression or changing phenotypes in response to environmental conditions [18–21].

Two major forces can affect gene flow between planktonic populations: abiotic factors, including marine currents, and biotic factors. First, as planktonic species are transported passively and continuously by marine currents, we could expect that the “isolation-by-current” shapes the genetic structure of populations. Conversely, cosmopolitan, panmictic and/or unstructured species have been reported multiple times in Copepoda [18, 22–26], Collodaria [25], and Cnidaria [26]. Other studies have shown more complex patterns, with genetic structures mainly observed at the basin level in Copepoda [27]⁠, Pteropoda [28]⁠, diatoms [29]⁠, and Cnidaria [30] or at the mesoscale in Chaetognatha [31]⁠, Hexanauplia [32–34], Dinophyceae [35], and *Macrocystis pyrifera* [36]⁠. Given the complexity of oceanic processes, classical landscape genomics frameworks have been adapted [37] to better model the dispersion and marine currents on populations over the seascape. In seascape genomics, the “isolation-by-currents” replaced the “isolation-by-distance” effect [38]. In this context, modelling oceanic circulation at the macro- and meso-scales is a prerequisite for capturing water mass connectivity [38]⁠. Successful approaches using data derived from larval dispersal models have been used in fish and coral [39–41] ⁠ and the use of Lagrangian travel time estimates combined with genetic data has shown promising results in explaining gene flow [33, 36].

Simultaneously, changing environmental conditions may lead to selective pressure that counteracts the effect of dispersion induced by marine currents, leading to higher differentiation. Some good examples are temperature-driven genetic structures from bacteria to cnidaria [15, 30, 42] ⁠ and the effect of salinity and silicate in diatoms that can even favor speciation in estuaries [43–45]. Biotic drivers based on competition and coevolution have also been reported to shape evolution [46]⁠. These findings enhanced our understanding of plankton connectivity, but they focused on documented species with reference sequences or often used few molecular markers, such as mitochondrial (COI) or ribosomal genes (16S, 18S, 28S), and/or were restricted to mesoscale sampling.

Advances in environmental genomics realized by shotgun sequencing offer a new perspective for the population genomics of marine plankton species based on metagenomic data. Diversity in ocean microorganisms can now be better-understood, thanks to ambitious expeditions [47, 48]. Particularly, *Tara* Oceans data provide a unique dataset from many locations in all oceans worldwide, enabling global approaches to investigate plankton [49–52], but blind spots in terms of taxonomy or function are still an obstacle for further analyses due to the lack of reference genomes or transcriptomes, especially for eukaryotes. The first approach to address this issue relies on the use of metagenome-assembled genomes (MAGs) that enable the retrieval of numerous lineages from metagenomic samples, especially small-sized genomes found in viruses, prokaryotes, and protists [45, 49, 53–56]. The second method is single-cell sequencing after flow cytometric sorting, which allows genome reconstruction of small eukaryotic species [57].

An alternative method for studying plankton population genomics has been proposed based on single nucleotide polymorphisms (SNPs) calling directly from metagenomic data using an assembly-free strategy [58]. The latter uses DiscoSNP +  + [59], a SNP calling tool applied directly to raw high-throughput sequencing data without assembly. Its application to *Tara* Oceans metagenomic data generated 18 million SNPs and a proof of concept of their utility and robustness for population genomics has been demonstrated on the epilagic copepod *Oithona nana* using its genome as a reference to relocating SNPs [58]. SNPs can also be directly clustered by species to bypass the use of genome references. To perform this, metaVaR was developed and applied as a proof-of-concept to simulated and real metagenomic datasets [60]. This approach allows the profiling of the genomic differentiation of several species separately and opens gates for new investigations.

Here, we propose to study plankton connectivity from a holistic point of view, using metagenomic data extracted from samples gathered during *Tara* Oceans expeditions in the Mediterranean Sea, Atlantic Ocean, and Southern Ocean. We clustered the 18e^6^ SNPs into 113 taxa that may correspond to complexes of closely related species. Minor allele frequencies of each species were used to estimate genomic differentiation using pairwise *F*_ST_. The genomic distances were modelled with environmental parameters, including Lagrangian travel times between sampling sites [61] to estimate the relative contribution of environmental factors, especially marine currents, to the genetic connectivity of plankton populations.

## Results

### Taxonomy and biogeography of 113 plankton species based on reference-free SNPs

**Fig. 1:**
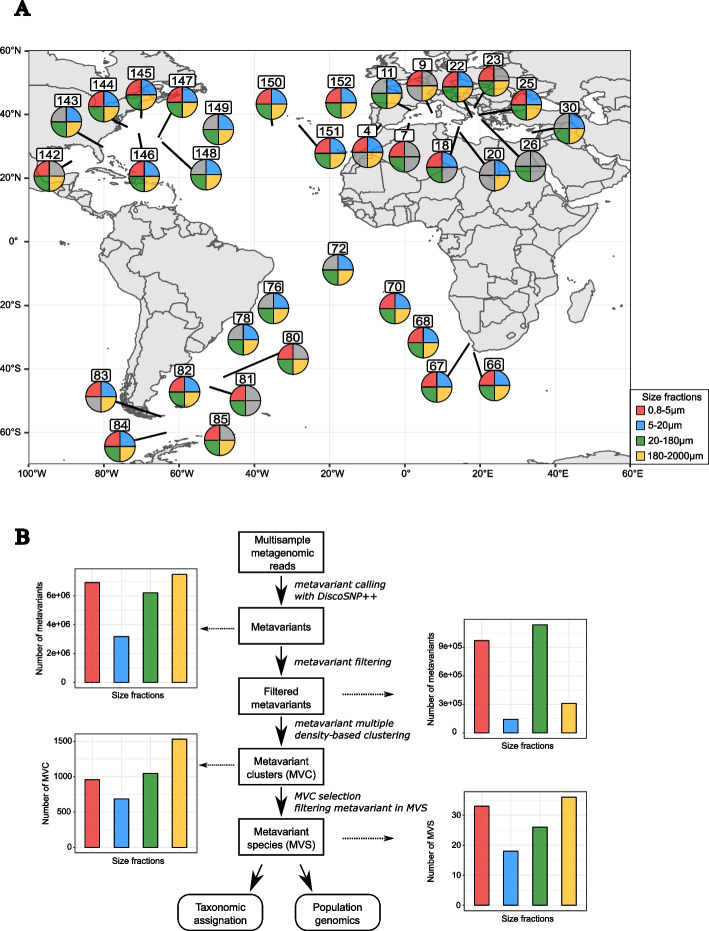
Clustering of SNPs by taxon from the metagenomic dataset of *Tara* Oceans. **A** Worldmap showing the locations of the 35 *Tara* Oceans stations used in the study. Each circle is divided into four parts, depending on the detection of plankton taxa by SNPs clustering. Grey colour indicates that no species were retrieved. **B** Pipeline to cluster SNPs by species using metaVaR, with additional statistics by size fraction. From top to bottom: number of SNPs before and after filtering, number of SNPs clusters detected, and number of plankton taxa finally selected

**Fig. 2:**
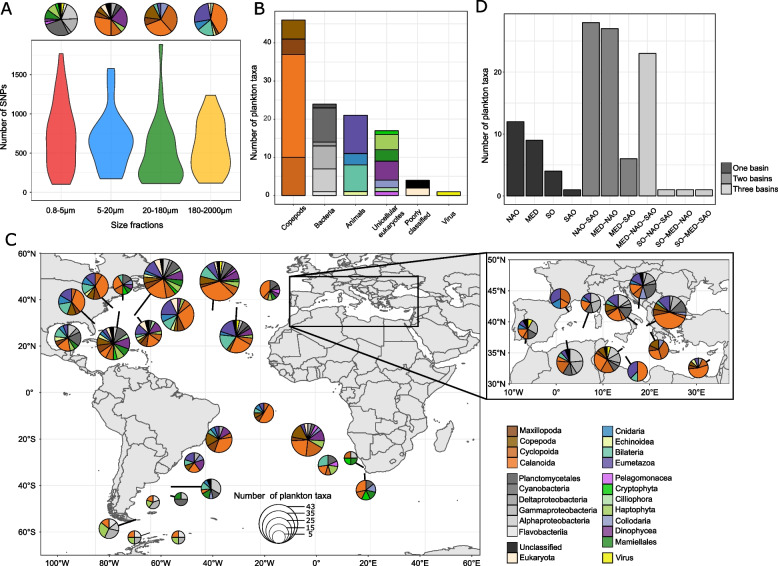
Taxonomy and biogeography of the plankton species. **A** Distribution of the number of SNPs for each size fraction. On the top, pie charts represent the taxonomic composition of each size fraction. **B** Number of taxa assigned to the six wider taxonomic groups. **C** Number of plankton taxa according to the basins they were detected in: Northern Atlantic Ocean (NAO), SAO (Southern Atlantic Ocean), SO (Southern Ocean), and MED (Mediterranean Sea). **D** World map showing the number of taxa of each taxonomic group for each *Tara* station. The size of the circles corresponds to the number of species detected in each station. The colours of taxonomic groups are indicated on the bottom right of the panel

We used over 18e^6^ SNPs called using a reference-free approach generated from 114 metagenomic samples collected from 35 *Tara* stations (Fig. 1A). The SNPs were clustered into groups SNPs belonging to the same taxon, and the minor allele frequencies of each SNPs were computed by population (Fig. 1B, Supplementary Table S1). Most of the SNPs harbouring sequences used for taxonomic assignment did not show any signal due to lack of planktonic data in the public databases. However, we assigned 113 taxa showed various lineages spanning all plankton trophic levels with a predominance of Hexanauplia (46 taxa), Bacteria (24 taxa), and Eumetazoa (21 taxa, comprising three Cnidaria and one Echinodea) (Fig. 2A and B). Among the Bacteria, we found nine Cyanobacteria, with eight taxa assigned to *Synechococcus* and one assigned to *Prochlorococcus*. Other notable eukaryotic taxa include Dinophycea (5), Haptophyta (4), Mamiellales (3), Collodaria (2), Ciliophora (2), Cryptophyta (1), and *Pelagomonadaceae* (1). Only four taxa presented very poor assignment (unclassified or eukaryotes) and one virus. In Mamiellales, two species were identified as *Bathyccocus prasinos* and were related to previously observed results from *Tara* Oceans (Supplementary Table S2). The number of SNPs per taxon ranged from 114 to 1,767. As expected, bacteria dominated the smaller size fractions, and Eumetazoan (Cnidaria, Copepods, and other Bilateria) were found in the larger size fractions. Most taxa (95. 84%) were present at four to six stations, with a maximum of eight stations (Supplementary Figure S1). The number of taxa per station showed an important variation (Fig. 2C), from four to 43 taxa (in TARA_67/81/84/85 and TARA_150, respectively). Notably, stations from the Southern Ocean (TARA_82 to 85) contained fewer taxa (from 4 to 7), with four taxa (Gammaproteobacteria, Haptophyta, Flavobacteria, and Calanoida) being present solely in the Southern Ocean (SO). Finally, 36 taxa were present in only one basin, while the majority (80 taxa) occurred in the Northern Atlantic Ocean (NAO) and one other basin (Fig. 2D).

### Global view of plankton genetic connectivity in the surface layer

**Fig. 3:**
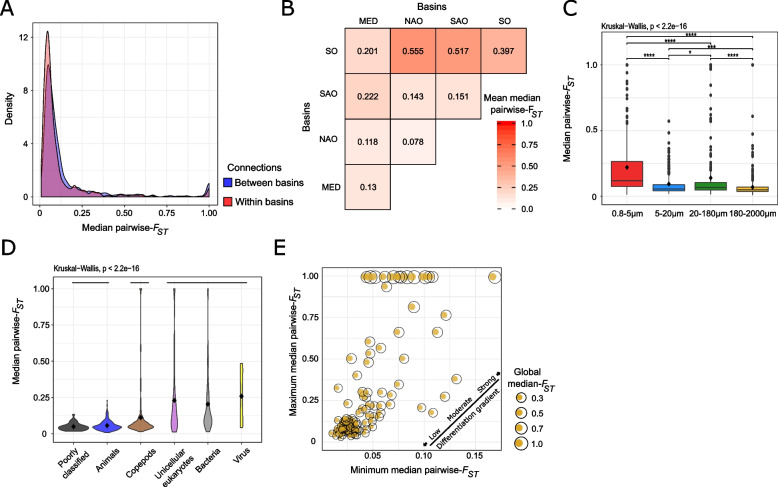
Global view of genomic differentiation of plankton populations. **A** Distributions of the 113 taxa’s pairwise *F*_ST_ matrices. In red, pairwise *F*_ST_ of populations belonging to the same basin; in blue to different basins. **B** Pairwise *F*_ST_ matrix between basins. The values represent the mean of all the median-*F*_ST_ between stations regrouped according to the basin they belonged to. **C** Distributions of the taxa’s median pairwise *F*_ST_, according to their size fractions. Black diamonds correspond to the mean of the distributions. The bars on the top correspond to the comparisons done by pairwise Wilcoxon tests (*p*-values: * < 0.05, ** < 0.01, *** < 0.001, **** < 0.0001) **D** Distributions of the taxa’s median pairwise *F*_ST_, according to their taxonomic group. Black diamonds correspond to the mean of the distributions. Each bar corresponds to taxonomic groups displaying no significant differences. **E** Scatter plot, each dot is a taxon. The size of each dot reflects the global median *F*_ST_ of the taxa’s *F*_ST_ distribution (i.e., *F*_ST_ computed over all the populations of a taxon)

Pairwise *F*_ST_ was used to estimate the population genomic differentiation. As expected, the global population genetic connectivity was higher within than between basins for each size fraction, either separately or together (Fig. 3A). Overall, taxa occurring in NAO presented moderate differentiation from their populations in the Mediterranean Sea (MED) and the Southern Atlantic Ocean (SAO) (0.118 and 0.143, respectively) (Fig. 3B). SAO and MED presented relatively high differentiation (0.222). Finally, this analysis underlined the important global differentiation of the SO from other basins (0.201–0.555), but also a high differentiation within the SO (0.397). The population genetic connectivity was significantly different between size fractions (Kruskal–Wallis, *p*-value < 0.05), being higher in the 180–2000 µm and lower in 0.8–5 µm and (Fig. 3C). Population differentiation between the six larger taxonomic groups was related to the body size of the lineages, with differentiation being relatively lower in copepods and other animals than in unicellular eukaryotes, bacteria, and viruses (Fig. 3D). Figure 3E shows a large spectrum of population genomic differentiation patterns, with a maximum median pairwise-*F*_ST_ between 0.03 and 1. Extreme cases with a median pairwise *F*_ST_ of 1 were observed for 13 taxa, and a global *F*_ST_ distribution strongly shifted to 1, as exemplified by Collodaria species (15_200_2) (Supplementary Figure S2). These 13 taxa illustrate the fact that our approach generates a non-negligible proportion of clusters of SNPs corresponding to complexes of closely-related species.

### The relative role of the environmental actors in population genetic connectivity

**Fig. 4:**
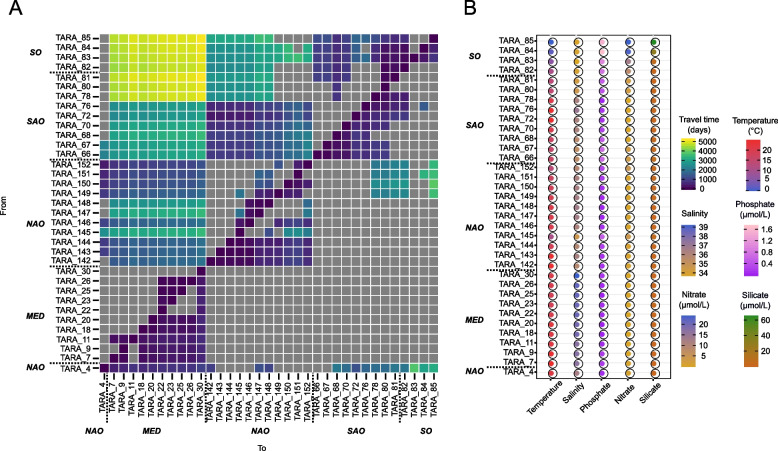
Lagrangian travel times and other environmental parameters. **A** Minimum times retained for analyses. In grey, asymmetric times were not the minimum, thus the matrix accounts for the “direction” of currents between stations. **B** Measures of temperature, salinity, nitrate, phosphate, and silicate extracted from World Ocean Atlas (WOA) for the 35 *Tara* stations. On the right, colour scales for each parameter. For the world map of *Tara* stations, see Supplementary Figure S3

**Fig. 5:**
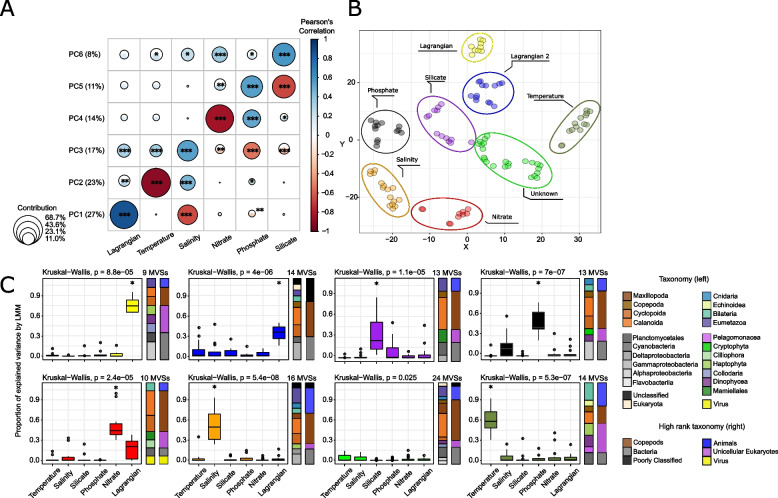
Variation partitioning of genomic differentiation for 113 plankton taxa. **A** PCA was performed on the proportion of variation explained by each parameter over the 113 plankton taxa. The colour corresponds to Pearson’s correlation between the coordinates of species for a component and the variation explained by the parameters (*p*-values: * < 0.05, ** < 0.01, *** < 0.001, **** < 0.0001). The size of the circles represents the relative contribution (i.e. the ratio of the variable cos^2^ on the total cos^2^ of the component) of each variable to each component. **B** t-SNE and k-means (K = 8) clustering. Each dot represents a taxon. Each colour corresponds to a defined cluster obtained by k-means. The names of the clusters are linked to the following figure **C** Distributions of variation are explained by each factor by cluster and the taxonomic composition of each cluster. The boxplot colours are the same as in the previous figure. The asterisk * on the top of boxplots corresponds to parameters that significantly contribute the most to the genomic differentiation of the taxa included in the cluster, according to a pairwise Wilcoxon test (*p*-value < 0.05)

We modelled the pairwise-*F*_ST_ of each taxon as the response variable explained by six environmental factors Lagrangian times (Fig. 4A), temperature, salinity, nitrate, silicate, and phosphate (Fig. 4B) using a linear mixed model (LMM). The fixed part of the explained variance was low for each taxon, ranging from 0 to 14% and was not further analysed (Supplementary Table S1). Among all tested environmental variables, Lagrangian travel time, temperature and salinity were the major contributors to genomic differentiation and were highly correlated to the first three components (67% explained variance) (Fig. 5A). The variance contributions of nitrate, silicate and phosphate respectively followed the last three components.

The taxa were then clustered into eight groups using k-means based on their t-SNE coordinates (Fig. 5B). We identified the most important variables for each taxon in each cluster (Fig. 5C). Two clusters were linked to Lagrangian travel times, labelled as “Lagrangian” (14 species) and “Lagrangian 2” (13), the latter exhibiting a lower variance explained by Lagrangian. The largest cluster contained 24 taxa but was not linked to any parameter. The other taxa clusters were linked to a single environmental parameter, such as salinity for 16 taxa and temperature, silicate, phosphate and nitrate for 14, 13, 13 and 10 taxa, respectively. The clusters “Lagrangian”, “Temperature” and “Salinity” presented clear differences between their respective drivers compared to the other parameters (Fig. 5C). The clusters “Phosphate” and “Silicate” showed a wider distribution of their respective driver among the taxa they contained, with respectively salinity and phosphate sharing a high proportion of explained variance. The “Nitrate” cluster also regrouped taxa for which a non-negligible part of variance was explained by Lagrangian travel time. Each cluster contained taxa assigned to almost all taxonomic groups and presented no particular visual enrichment (Fig. 5C). This absence of enrichment was clearer in copepods, which constituted most species (Fisher’s Exact Test *p*-value = 0.348).

**Fig. 6:**
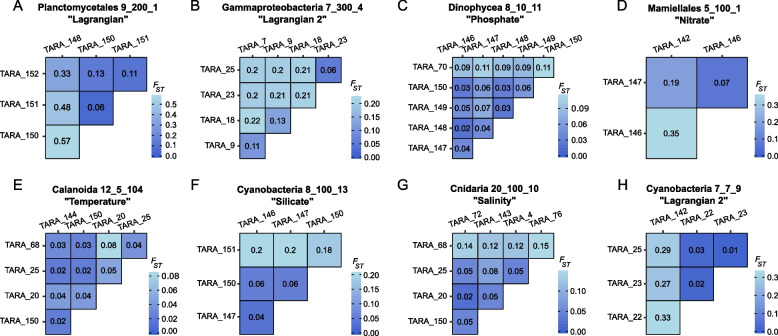
Examples of genomic differentiation. From **A** to **H** Pairwise *F*_ST_ matrices of plankton taxa mentioned in the respective titles. For each title are mentioned: the taxonomic assignment, the species ID, and the name of the cluster the species belongs to (clusters based on the abiotic parameters driving the population connectivity)

**Fig. 7:**
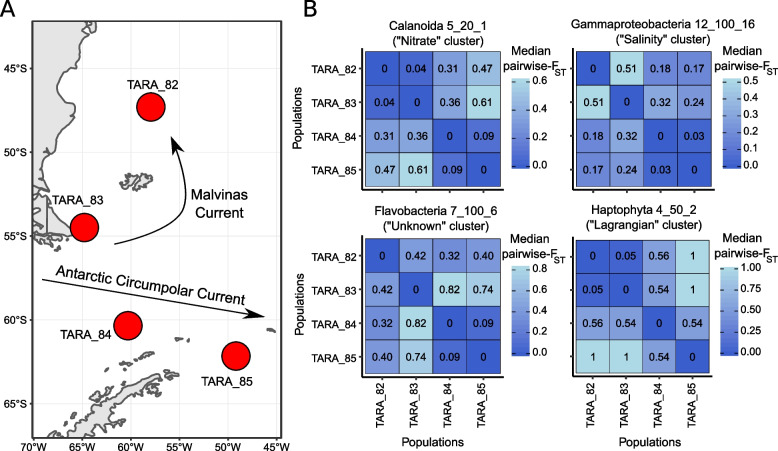
Genomic differentiation in Southern Ocean. **A** Map localizing TARA_82, 83, 84, and 85. The two arrows correspond to the trajectories of currents, based on Lagrangian trajectories, travel times, and literature **B** Pairwise *F*_ST_ matrices of the four species specifically occurring in the Southern Ocean

Among the taxa belonging to the “Lagrangian” cluster, we observed five taxa present in the Mediterranean Sea and Southern Atlantic and one in the Northern and Southern Atlantic. Two taxa were restrained to a single basin, the Southern Ocean and Northern Atlantic. Notably, the latter, Planctomycetales (9_200_1) shows population genomic differentiation linked to local marine barriers, with the population from TARA_148 being more isolated from the others (TARA_150, 151 and 152) (Fig. 6A). Another example of within–basin differentiation concerns the Mediterranean Gammaproteobacteria 7_300_4 from the “Lagrangian 2” cluster. The genomic differentiation clearly shows a pattern correlated to the Mediterranean marine currents (Fig. 6B), with less connectivity between TARA_7, 9 and TARA_23, 25, 18. In the SO, Gammaproteobacteria (12_100_16), Flavobacteria (7_100_6), Haptophyte (4_50_2) and Calanoid (5_20_1) were observed at stations TARA_82, 83, 84 and 85, where two main currents in the area were spotted: the Malvinas Current and the Antarctic Circumpolar Current (ACC) (Fig. 7A, Supplementary Figure S4). These four taxa presented among the highest global median *F*_ST_ (0.35 to 0.84) and revealed very low connectivity between their populations (Fig. 7B). Particularly, Haptophyta species present genomic differentiation linked to both the ACC and the Malvinas Current.

Certain taxa display a clear correlation between their population genetic connectivity and a single environmental parameter that differs from the marine current. For example, in the “Phosphate” cluster, the Dinophyceae (8_10_11) population from TARA_70 was more isolated from the other NAO populations and the TARA_70 site is also characterized by a higher phosphate concentration (0.264 µmol.L^−1^ against 0.031–0.106 µmol.L^−1^) (Fig. 6C). In the “Nitrate” cluster, the populations of a Mamiellales taxon (5_100_1) from TARA_146 and TARA_147 were highly connected and this correlated with the variation of nitrate concentration (Fig. 6D). In the “Temperature” cluster, the widely distributed Calanoida species (12_5_104), detected in the MED, NAO, and SAO presented a relatively higher genetic distance between populations from TARA_20 and 68 (*F*_*ST*_ = 0.08) (Fig. 6E) and was linked to a higher temperature difference. We observed genomic differentiation along a silicate gradient for a cyanobacteria (8_100_13), showing high isolation of the TARA_151 population compared to populations from TARA_146, 147 and 150 (Fig. 6F). The genetic isolation of TARA_151 was correlated to a higher concentration of silicate in the North-East Atlantic.

We also found a few taxa with a large proportion of the genomic differentiation explained by two factors, as for a Cnidaria (20_100_10) from the “Salinity” cluster, with temperature being also an important explaining factor (Fig. 6G). It was also the case for a cyanobacteria (7_7_9) from “Lagrangian 2” cluster which presented a high genomic differentiation between MED and NAO (Fig. 6H) that correlates with both Lagrangian travel times and salinity, the Mediterranean Sea presenting higher salinity than NAO.

## Discussion

### Reference-free approach for non-model species population genomics

Thanks to our approach exploiting metagenomic data, the population connectivity was reconstructed for planktonic eukaryote taxa representing the different trophic levels of the epipelagic layer of oceans and enabled a realistic overview of the population structures of marine planktonic species lacking reference sequences. With hundreds of variants per taxon, we drew the silhouette of population structures across four oceanic areas using more markers than previous studies often based on few genetic markers, few samples, and limited to small geographic areas. It must be noted that for each taxon, most sequences did not show any taxonomic signal, an observation already made in other studies using *Tara* Oceans data [50, 52]. The level and quality of taxonomic assignment are both due to a lack of references in databases and the short length of the sequences supporting the variants, reducing the chance of matching a reference with an acceptable coverage and having a taxonomic assignment with a high resolution. Notwithstanding these technical limitations for taxonomic annotation, four notable taxonomic groups have been described and could be related to previous observations. First, we were able to detect a virus from the order Caudovirales which probably belongs to the bacteriophage family of Myoviridae. These viruses are known to be abundant compared to other viruses in oceans [62]⁠, notably infect Cyanobacteria (i.e., *Prochlorococcus* and *Synechococcus*), and constitute most viral populations in GOV 2.0 [63]⁠. Second, two Cyanobacteria (15_500_9 and 7_20_37), probably belonging to the *Synechococcus* genus, were detected in the same locations in the Mediterranean Sea, with clear *F*_ST_ unimodal distributions (Supplementary Figure S2) and could be related to the ecotypes of Mediterranean *Synechococcus* [64]⁠. Third, in protists, two species corresponding to Mamiellales (6_5_14 and 9_500_10) are respectively located in *Tara* stations where *Bathycoccus prasinos* and *Bathycoccus spp. TOSAG39–1* were the most abundant (Supplementary Table S2), as described in a previous study using *Tara* Oceans metagenomic dataset [65]⁠. Finally, copepods formed the largest group, with a predominance of calanoids over cyclopoids. Numerous copepods were expected considering their high abundance in oceans [66, 67] and good representation in the *Tara* Oceans dataset. A limitation of our approach was the presence of a cluster of SNPs predicted to belong to a single taxon but harbouring extreme pairwise-*F*_ST_ values, showing that some validated clusters of SNPs may refer to a complex of closely related species, as previously described for the cosmopolitan copepod *Oithona similis* [68]. While the reference-free approach provides interesting results, the lack of reference sequences does not allow downstream analyses to provide functional annotation of the SNPs. Future reference-based methods, including MAGs or newly built genome assemblies, will greatly help to capture more polymorphisms, refine taxonomical assignment, and allow the identification of genes and their related functions impacted by nucleotide polymorphisms.

We showed that populations of smaller organisms, such as protists and bacteria, are more structured than those of zooplankton. These first two groups of organisms are not characterized by the same range of demographic parameters, such as population size, dispersal capacity, or generation time, leading to very different effects on their evolution. Moreover, these taxa experienced radically different demographic histories, limiting the comparisons from the use of *F*_ST_, an estimate affected by the population effective size, described as large among plankton organisms in the few studies that estimated this parameter [14, 69, 70].

### Relative effects of environment and currents on macro-scale population genetic connectivity of plankton

Over 113 plankton taxa, Lagrangian travel time, salinity and temperature were the most important tested genomic differentiation drivers, while nitrate, silicate and phosphate had a relatively lower impact and this does not seem to be clade-specific. The effect of Lagrangian travel time on population differentiation illustrates the role of ocean currents and seascape dynamics in population genetic connectivity and validates the isolation-by-current hypothesis for a non-negligible number of plankton species. Here, we showed that populations belonging to different basins tend to be more differentiated than populations located in the same basin, which could be easily explained by relatively smaller connections within basins than between basins. While this trend has been observed several times [28, 71, 72], interesting patterns of population genetic connectivity remain between the basins. We observed the central role of the NAO, which connects its populations to both MED and SAO, and a slightly lower connection between MED and SAO. Some plankton populations from the SO were isolated from the other basins. This situation has already been observed in the copepod *Metridia lucens* [73]⁠, as well as important differentiation within the SO. This area is characterized by differences in environmental conditions, and compared to the rest of the basins, with higher silicate, nitrate and phosphate concentrations on one hand, and lower salinity and temperature (Fig. 4B). Additionally, water masses are driven over thousands of kilometers by the complex Antarctic Circumpolar Current (ACC) [74]⁠, which could favour long-range gene flow around the Antarctic. The Lagrangian data traced the northward Malvinas current (an ACC branch), which mixes warm water masses from the Brazil current with cold waters of the ACC in the Brazil–Malvinas confluence [75]⁠, possibly favouring the isolation of plankton populations in the south of this area. This specific environment could explain why these species are both specific to the Austral *Tara* stations and are highly differentiated.

Salinity and temperature affect biogeography, community composition and population structure [15, 28, 44, 51, 76]. The role of nutrients such as nitrate [77], silicate [25, 78, 79]⁠, and phosphate [80] in marine microorganism metabolism and diversity has been well studied, but their impact on the population genetic structure has never been investigated at this scale [81–83]. A large part of the genomic differentiation could not be explained in this study, suggesting missing parameters. The absence of key physicochemical parameters, such as metals [21, 84], sulfur [85]⁠ and pH [19] could also enhance our understanding of plankton genomic differentiation. The contribution of biotic interactions between trophic levels, such as zooplankton grazing on phytoplankton, should also be examined [86].

### A holistic view of plankton connectivity as a mosaic

By combining population genomics with environmental factors and seascape dynamics, we identified planktonic species groups with genomic differentiation driven by the same factors. These different groups of species allowed for the sketching of a mosaic of connectivity patterns in the seascape. This mosaic is underlined by the diversity of environmental conditions influencing the differentiation and shows that the living range of species is not correlated to their population structure, that is, cosmopolitan species do not necessarily present an absence of population structure and species with populations present in close locations can exhibit high differentiation (such as SO). Thus, we showed how population genomic analyses at different trophic levels are important to decipher the connectivity of plankton and can be complementary to the traditional metabarcoding approach that fails to quantify the connectivity and intra-species structure patterns. The next step would be to better capture the relative effects of evolutionary forces acting on plankton genomes, such as genetic drift and selection. Haplotype data could resolve this question, but in the framework of metagenomics, the latter remains a technical and computational challenge.

Global warming and its impact on ocean climate are expected to have a significant impact on marine plankton biogeography by restructuring plankton assemblages [87]. In this context, the relative role of seascape dynamics and environmental factors in plankton population genetic connectivity can be expected to shift towards a major role of temperature. Our ability to model plankton adaptation and evolution at the molecular level, in response to global warming, will permit better projections of future plankton biogeography.

## Material and methods

### Single nucleotide polymorphisms from *Tara* Oceans metagenomic data

We used a set of 18e^6^ SNPs produced in a previous study [58]. SNPs were detected from metagenomic data generated from 35 *Tara* Oceans sampling sites corresponding to four distinct size fractions (0.8–5 µm, 5–20 µm, 20–180 µm, and 180–2000 µm) from the water surface layer, for a total of 114 samples (Fig. 1A). For further analyses, *Tara* stations were separated into four groups corresponding to the basins they belonged to: the Mediterranean Sea (MED; TARA_7 to TARA_30), Northern Atlantic Ocean (NAO; TARA_4, TARA_142 to TARA_152), Southern Atlantic Ocean (SAO; TARA_66 to TARA_81), and Southern Ocean (SO; TARA_82 to TARA_85). The full protocols for sampling, extraction and sequencing have been detailed in previous studies [88, 89]. All maps in the figures of the current study were generated with the rnaturalearth R package (https://github.com/ropensci/rnaturalearth).

### Clustering of intra-species SNPs

To identify taxon specific SNPs, we used metaVaR version v0.2 [60]. We discarded SNPs called from low-covered loci, repeated regions that present very high coverage, and SNPs from loci with non-null coverage in less than four samples. This was performed using metaVarFilter.pl with parameters -a 5 -b 5000 -c 4. The filtered SNPs were clustered based on the covariation of their loci depth of sequencing coverage using multiple density-based clustering instances [90, 91], a total of 187 couples of parameters epsilon and minimum points (ε, MinPts) were tested with epsilon ε = {4, 5, 6, 7, 8, 9, 10, 12, 15, 18, 20} and MinPts = {1, 2, 3, 4, 5, 6, 7, 8, 9, 10, 20, 50, 100, 200, 300, 400, 500}. This clustering generated a set of clusters for each parameter couple (Supplementary Figure S5). To retain taxon specific SNPs, we selected non-overlapping clusters, that is, clusters sharing no SNPs and maximizing a score based on the distribution of the sequencing depth of coverage of the loci (expected to follow a negative binomial distribution). The selection was performed by applying a maximum-weighted independent set algorithm to the scored clusters. To avoid any allele frequency bias due to low depth of coverage, we selected only loci with a depth of coverage between 8 × and the maximum expected depth of coverage in all populations, based on the empirical depth of coverage distribution. Finally, only clusters with at least 100 SNPs, for which at least three samples presented a median depth of coverage over 8 × were retained, leading to a final set of 113 clusters corresponding to species (or complex of closely related species). For each species, we generated an allele frequency matrix for each biallelic locus.

### Taxonomic assignment of species

Taxonomic assignment of each species was performed using three different methods (Supplementary Figure S5). For the first method, the short sequences supporting the variants (generated by DiscoSNP + +) were mapped on the downloaded NCBI non-redundant database with diamond v0.9.24.125 [92]⁠ using blastx and parameter -k 10, and the results were filtered based on the E-value (< 10^–5^). The taxonomic ID and bit scores of each match were maintained. A fuzzy Lowest Common Ancestor (LCA) (see https://github.com/institut-de-genomique/fuzzy-lca-module) method was used to assign a taxonomy to each sequence using bitscore as a weight with -r 0.67 (i.e. taxa covering at least 67% of all bitscores) and -ftdp options. The highest phylogenetic rank was retained as the best assignment for each sequence. For the second method, the sequences were mapped using the blastn algorithm implemented in diamond on MATOU, a unigen catalog based on *Tara* Oceans metatranscriptomic data [50]⁠, and the last method involved mapping the sequences on the MMETSP transcriptomic database [93]⁠. The species (or a complex of closely related species) were assigned to the most probable taxon to offer three taxonomic assignment levels, from the most precise to the widest (Supplementary Table S1). The final set of taxa was first grouped into 24 taxonomic groups and finally merged into six reliable wider groups (Viruses, Bacteria, Unicellular Eukaryotes, Copepods, and other animals, and poor classification) (Fig. 2B).

### Population genomics analysis

To investigate the genomic differentiation of each taxon, *F*_ST_ was used and computed for each variant as follows: *F*_ST_ = $\frac{{\sigma }^{2}}{\overline{p}\left(1-\overline{p}\right)}$, where $\overline{p}$ and ${\sigma }^{2}$ are, respectively, the mean and variance of minor allele frequency across the considered populations [94]. Two *F*_ST_ calculations were performed, the global *F*_ST_ was calculated using among all populations, allowing the analysis of the global *F*_ST_ distribution. Then, a pairwise *F*_ST_ was calculated between the populations and the median pairwise *F*_ST_ was retained as a measure of genomic differentiation between the populations. We tested the effect of oceanic basins, taxonomy, and size fraction on the genomic differentiation of each species using a Kruskal–Wallis test. When the test was significant (*p* < 0.05), multiple comparison Wilcoxon tests were performed between groups. To estimate the genetic connectivity between and within basins, we regrouped *Tara* stations based on their locations (i.e., MED, NAO, SAO and SO) and computed the mean *F*_ST_ between and within basins.

### Lagrangian travel time estimation and environmental data

To estimate Lagrangian transport, we used a method based on drifter data [61] to compute the travel time of the most likely path between *Tara* stations back and forth. We used the public database of the Global Drifter Program (GDP), managed by the National Oceanographic and Atmospheric Administration (NOAA) (https://www.aoml.noaa.gov/phod/gdp/), which contains information on drifters ranging from February 15, 1979, to September 31, 2019. We extracted the data for both drogued and undrogued drifters (i.e., drifters that lost their socks) to maximize the information. No drifters have been observed to exit the Mediterranean Sea through the Strait of Gibraltar. Therefore, to avoid missing data, we arbitrarily added 100 years to the travel times of pathways out of the Mediterranean Sea over the Strait of Gibraltar and added one year to the pathways going into the Mediterranean Sea, based on previous models of surface water [95, 96]. We used 450 rotations within the method to reduce the reliance on travel times on the grid system used. Two travel times are obtained by the method for each pair of stations, back and forth, resulting in an asymmetric travel time matrix between all possible station pairings. For our analyses, we retained only the minimum of the two travel times.

Environmental variables corresponding to the 35 selected *Tara* stations were extracted from the World Ocean Atlas public database (https://www.nodc.noaa.gov/OC5/woa13/woa13data.html) for the period 2006–2013 on a 1° × 1° grid, covering the dates of *Tara* Oceans expeditions. The following parameters were obtained: temperature (°C), salinity (unitless), silicate (µmol.L^−1^), phosphate (µmol.L^−1^), and nitrate (µmol.L^−1^) (Supplementary Figure S7).

### Variation partitioning of the genomic differentiation

To estimate the relative contribution of environmental parameters and Lagrangian travel time to the variance of the genetic connectivity, a LMM was applied using the R package MM4LMM [97]⁠. The model applied was as follows: ${Y}_{FST}=\mu +Zu+\varepsilon$, where *Y*_*FST*_ is the vector of observations of *F*_ST_ values with a mean *µ, Z* is a known matrix of parameters relating the observations* Y*_*FST*_ to *u*, is a vector of independent random effects of zero mean, and *ε* is a vector of random errors of 0 means and covariance matrix proportional to the identity (white noise). For each pairwise *F*_ST_ matrix, the corresponding matrix of the minimum Lagrangian travel time is retrieved. Temperature, salinity, silicate, phosphate, and nitrate measurements were extracted for all the stations where the plankton species were present, and a Euclidean distance was computed between the stations for each of these parameters. The LMM was then applied to pairwise *F*_ST_ values using the five environmental distances and Lagrangian travel times after scaling, adding a variance of 1 for each explicative variable. We considered these parameters as the independent variables. As a result, an estimate of the contribution of each parameter to the total variance of the pairwise *F*_ST_ was obtained. Additionally, a fixed effect and proportion of unexplained variance were retrieved. After *F*_ST_ variance decomposition, two principal component analyses (PCA) were performed. The first was performed on the variance explained by the six variables and the unexplained part of the variance over the 113 species. From this PCA, the unexplained *F*_ST_ variance (Supplementary Figure S8) was high in most species, strongly contributing to the first component (37% explained variance). For clarity, a second PCA was performed by removing the unexplained part of the variance. For both PCAs, the correlation of the variables with the components and the contribution (i.e., the ratio of cos^2^ of each variable to the total cos^2^ of the components) of the variables to the components were extracted. PCAs were performed using the FactoMineR v2.3 R package [98].

### Identification of species with similar environmental parameters-driven genetic connectivity

To identify taxa sharing similar environmental parameters that drive their genetic connectivity, the variance explained by each factor was used with dimensional reduction through t-distributed stochastic neighbour embedding (t-SNE) using the Rtsne R package [99] with a perplexity of 5 and 5,000 iterations, and we extracted the taxa coordinates. Subsequently, k-means clustering was performed to identify taxa with common patterns of explained variance, with K = 8 based on the observation of the t-SNE point density. To identify which set of parameters drives the differentiation of a cluster, we compared the distributions of the explained variance of each parameter within the cluster using the Kruskal–Wallis and Wilcoxon paired tests (*p* < 0.05).

### Supplementary Information

**Additional file 1.**

**Additional file 2: Supplementary Figure S1.** Occurrence of species. Species are noted “MVS” for metavariant species. **Supplementary Figure S2.** Distributions of pairwise-*F*_*ST*_ by species. **Supplementary Figure S3.** Lagrangian estimates matrices. **Supplementary Figure S4.** Lagrangian trajectories for stations of Southern Ocean. **Supplementary Figure S5.** SNPs clustering with metaVaR. **Supplementary Figure S6.** Overview of the taxonomic assignment procedure. **Supplementary Figure S7.** Environmental parameters maps. **Supplementary Figure S8.** Principal component analysis of the contribution of environmental parameters to the genomic differentiation of plankton species. **Supplementary Table S2.** Species assigned to *Bathycoccus*.

## Data Availability

All the data is available on github at: https://github.com/rlasojad/Metavariant-Species.

## References

[1] Smith ADM, Brown CJ, Bulman CM, Fulton EA, Johnson P, Kaplan IC (2011). Impacts of fishing low-trophic level species on marine ecosystems. Science.

[2] Worm B, Barbier EB, Beaumont N, Duffy JE, Folke C, Halpern BS (2006). Impacts of biodiversity loss on ocean ecosystem services. Science.

[3] Bucklin A, Ortman BD, Jennings RM, Nigro LM, Sweetman CJ, Copley NJ (2010). A “Rosetta Stone” for metazoan zooplankton: DNA barcode analysis of species diversity of the Sargasso Sea (Northwest Atlantic Ocean). Deep-Sea Res Part II Top Stud Oceanogr.

[4] Malviya S, Scalco E, Audic S, Vincent F, Veluchamy A, Poulain J (2016). Insights into global diatom distribution and diversity in the world’s ocean. Proc Natl Acad Sci U S A.

[5] Karlusich JJP, Ibarbalz FM, Bowler C (2020). Phytoplankton in the Tara Ocean. Annu Rev Mar Sci.

[6] Longhurst AR, Harrison WG (1989). The biological pump: profiles of plankton production and consumption in the upper ocean. Prog Oceanogr.

[7] Steinberg DK, Landry MR (2017). Zooplankton and the ocean carbon cycle. Annu Rev Mar Sci.

[8] Lima-Mendez G, Faust K, Henry N, Decelle J, Colin S, Carcillo F (2015). Determinants of community structure in the global plankton interactome. Science.

[9] Wassmann P, Reigstad M, Haug T, Rudels B, Carroll ML, Hop H (2006). Food webs and carbon flux in the barents sea. Prog Oceanogr.

[10] Beaugrand G (2002). Reorganization of North Atlantic marine copepod biodiversity and climate. Science.

[11] Guinder VA, Molinero JC. Climate change effects on marine phytoplankton. Marine Ecology in a Changing World. 2013:68–90.

[12] Norris RD (2000). Pelagic species diversity, biogeography, and evolution. Paleobiology.

[13] Collins S, Rost B, Rynearson TA (2014). Evolutionary potential of marine phytoplankton under ocean acidification. Evol Appl.

[14] Peijnenburg KTCA, Goetze E (2013). High evolutionary potential of marine zooplankton. Ecol Evol.

[15] Delmont TO, Kiefl E, Kilinc O, Esen OC, Uysal I, Rappé MS (2019). Single-amino acid variants reveal evolutionary processes that shape the biogeography of a global SAR11 subclade. eLife.

[16] Hellweger FL, Sebille EV, Fredrick ND (2014). Biogeographic patterns in ocean microbes emerge in a neutral agent-based model. Science.

[17] Ron R, Fragman-Sapir O, Kadmon R (2018). Dispersal increases ecological selection by increasing effective community size. Proc Natl Acad Sci U S A.

[18] Laso-Jadart R, Sugier K, Petit E, Labadie K, Peterlongo P, Ambroise C (2020). Investigating population-scale allelic differential expression in wild populations of *Oithona similis* (Cyclopoida, Claus, 1866). Ecol Evol.

[19] Lewis CN, Brown KA, Edwards LA, Cooper G, Findlay HS (2013). Sensitivity to ocean acidification parallels natural pCO2 gradients experienced by Arctic copepods under winter sea ice. Proc Natl Acad Sci U S A.

[20] Maas AE, Lawson GL, Tarrant AM (2015). Transcriptome-wide analysis of the response of the thecosome pteropod Clio pyramidata to short-term CO2 exposure. Comp Biochem Physiol Part Genomics Proteomics.

[21] Mackey KRM, Post AF, McIlvin MR, Cutter GA, John SG, Saito MA (2015). Divergent responses of Atlantic coastal and oceanic synechococcus to iron limitation. Proc Natl Acad Sci U S A.

[22] Kozol R, Blanco-Bercial L, Bucklin A (2012). Multi-gene analysis reveals a lack of genetic divergence between *Calanus agulhensis* and *C. sinicus* (Copepoda; Calanoida). PLoS One.

[23] Provan J, Beatty GE, Keating SL, Maggs CA, Savidge G (2009). High dispersal potential has maintained long-term population stability in the North Atlantic copepod *Calanus finmarchicus*. Proc R Soc B Biol Sci.

[24] Weydmann A, Coelho NC, Serrão EA, Burzyński A, Pearson GA (2016). Pan-Arctic population of the keystone copepod *Calanus glacialis*. Polar Biol.

[25] Biard T, Bigeard E, Audic S, Poulain J, Gutierrez-Rodriguez A, Pesant S (2017). Biogeography and diversity of Collodaria (Radiolaria) in the global ocean. ISME J.

[26] Stopar K, Ramšak A, Trontelj P, Malej A (2010). Lack of genetic structure in the jellyfish *Pelagia noctiluca* (Cnidaria: Scyphozoa: Semaeostomeae) across European seas. Mol Phylogenet Evol.

[27] Goetze E (2011). Population differentiation in the open sea: Insights from the pelagic copepod pleuromamma xiphias. Integr Comp Biol.

[28] Burridge AK, Goetze E, Raes N, Huisman J, Peijnenburg KTCA (2015). Global biogeography and evolution of cuvierina pteropods phylogenetics and phylogeography. BMC Evol Biol.

[29] Casteleyn G, Leliaert F, Backeljau T, Debeer AE, Kotaki Y, Rhodes L (2010). Limits to gene flow in a cosmopolitan marine planktonic diatom. Proc Natl Acad Sci U S A.

[30] Werner S, Gerhard J, Bruno S, Bernd S (2002). Speciation and phylogeography in the cosmopolitan marine moon jelly, Aure-lia sp. BMC Evol Biol.

[31] Peijnenburg KTCA, Fauvelot C, Breeuwer JAJ, Menken SBJ (2006). Spatial and temporal genetic structure of the planktonic *Sagitta setosa* (Chaetognatha) in European seas as revealed by mitochondrial and nuclear DNA markers. Mol Ecol.

[32] Edmands S (2001). Phylogeography of the intertidal copepod *Tigriopus californicus* reveals substantially reduced population differentiation at northern latitudes. Mol Ecol.

[33] Madoui M-A, Poulain J, Sugier K, Wessner M, Noel B, Berline L (2017). New insights into global biogeography, population structure and natural selection from the genome of the epipelagic copepod Oithona. Mol Ecol.

[34] Yebra L, Bonnet D, Harris RP, Lindeque PK, Peijnenburg KTCA (2011). Barriers in the pelagic: population structuring of *Calanus helgolandicus* and *C. euxinus* in European waters. Mar Ecol Prog Ser.

[35] Richlen ML, Erdner DL, McCauley LAR, Liberal K, Anderson DM (2012). Extensive genetic diversity and rapid population differentiation during blooms of *Alexandrium fundyense* (dinophyceae) in an isolated salt pond on cape cod, MA, USA. Ecol Evol.

[36] Alberto F, Raimondi PT, Reed DC, Watson JR, Siegel DA, Mitarai S (2011). Isolation by oceanographic distance explains genetic structure for *Macrocystis pyrifera* in the Santa Barbara Channel. Mol Ecol.

[37] Fontaine MC, Baird SJE, Piry S, Ray N, Tolley KA, Duke S (2007). Rise of oceanographic barriers in continuous populations of a cetacean: the genetic structure of harbour porpoises in Old World waters. BMC Biol.

[38] Riginos C, Crandall ED, Liggins L, Bongaerts P, Treml EA (2016). Navigating the currents of seascape genomics: how spatial analyses can augment population genomic studies. Curr Zool.

[39] Dalongeville A, Andrello M, Mouillot D, Lobreaux S, Fortin M-J, Lasram F (2017). Geographic isolation and larval dispersal shape seascape genetic patterns differently according to spatial scale. Evol Appl.

[40] Galindo HM, Pfeiffer-Herbert AS, McManus MA, Chao Y, Chai F, Palumbi SR (2010). Seascape genetics along a steep cline: using genetic patterns to test predictions of marine larval dispersal. Mol Ecol.

[41] Riginos C, Hock K, Matias AM, Mumby PJ, van Oppen MJH, Lukoschek V (2019). Asymmetric dispersal is a critical element of concordance between biophysical dispersal models and spatial genetic structure in Great Barrier Reef corals. Divers Distrib.

[42] De Luca D, Piredda R, Sarno D, Kooistra WHCF (2021). Resolving cryptic species complexes in marine protists: phylogenetic haplotype networks meet global DNA metabarcoding datasets. ISME J.

[43] Sjöqvist C, Godhe A, Jonsson PR, Sundqvist L, Kremp A (2015). Local adaptation and oceanographic connectivity patterns explain genetic differentiation of a marine diatom across the North Sea-Baltic Sea salinity gradient. Mol Ecol.

[44] Ueda H, Yamaguchi A, Saitoh SI, Sakaguchi SO, Tachihara K (2011). Speciation of two salinity-associated size forms of *Oithona dissimilis* (Copepoda: Cyclopoida) in estuaries. J Nat Hist.

[45] Nef C, Madoui M-A, Pelletier É, Bowler C (2022). Whole-genome scanning reveals environmental selection mechanisms that shape diversity in populations of the epipelagic diatom Chaetoceros. PLOS Biol.

[46] Smetacek V (2012). Making sense of ocean biota: how evolution and biodiversity of land organisms differ from that of the plankton. J Biosci.

[47] Karsenti E, Acinas SG, Bork P, Bowler C, Vargas CD, Raes J (2011). A holistic approach to marine eco-systems biology. PLoS Biol.

[48] Yooseph S, Sutton G, Rusch DB, Halpern AL, Williamson SJ, Remington K (2007). The sorcerer II global ocean sampling expedition: expanding the universe of protein families. PLoS Biol.

[49] Brum JR, Ignacio-espinoza JC, Roux S, Doulcier G, Acinas SG, Alberti A (2015). Ocean viral communities. Science.

[50] Carradec Q, Pelletier E, Silva CD, Alberti A, Seeleuthner Y, Blanc-Mathieu R (2018). A global ocean atlas of eukaryotic genes. Nat Commun.

[51] Sunagawa S, Coelho LP, Chaffron S, Kultima JR, Labadie K, Salazar G (2015). Structure and function of the global ocean microbiome. Science.

[52] Vorobev A, Dupouy M, Carradec Q, Delmont TO, Annamalé A, Wincker P (2020). Transcriptome reconstruction and functional analysis of eukaryotic marine plankton communities via high-throughput metagenomics and metatranscriptomics. Genome Res.

[53] Delmont TO, Gaia M, Hinsinger DD, Fremont P, Guerra AF, Eren AM, et al. Functional repertoire convergence of distantly related eukaryotic plankton lineages revealed by genome-resolved metagenomics. BioRxiv. 2020:2020.10.15.341214.

[54] Parks DH, Rinke C, Chuvochina M, Chaumeil PA, Woodcroft BJ, Evans PN (2017). Recovery of nearly 8,000 metagenome-assembled genomes substantially expands the tree of life. Nat Microbiol.

[55] Stewart RD, Auffret MD, Warr A (2018). Assembly of 913 microbial genomes from metagenomic sequencing of the cow rumen. Nat Commun.

[56] Delmont TO, Gaia M, Hinsinger DD, Frémont P, Vanni C, Fernandez-Guerra A (2022). Functional repertoire convergence of distantly related eukaryotic plankton lineages abundant in the sunlit ocean. Cell Genomics.

[57] Seeleuthner Y, Mondy S, Lombard V, Carradec Q, Pelletier E, Wessner M (2018). Single-cell genomics of multiple uncultured stramenopiles reveals underestimated functional diversity across oceans. Nat Commun.

[58] Arif M, Gauthier J, Sugier K, Iudicone D, Jaillon O, Wincker P (2019). Discovering millions of plankton genomic markers from the Atlantic ocean and the Mediterranean Sea. Mol Ecol Resour.

[59] Uricaru R, Rizk G, Lacroix V, Quillery E, Plantard O, Chikhi R (2015). Reference-free detection of isolated SNPs. Nucleic Acids Res.

[60] Laso-Jadart R, Ambroise C, Peterlongo P, Madoui MA. MetaVaR: introducing metavariant species models for reference-free metagenomic-based population genomics. PLoS One. 2020:1–17. 10.1371/journal.pone.0244637 PMC7773188 33378381

[61] O’Malley M, Sykulski AM, Laso-Jadart R, Madoui M-A. Estimating the Travel Time and the Most Likely Path from Lagrangian Drifters. J Atmos Oceanic Technol. 2021;38:1059–73.

[62] Sullivan MB, Huang KH, Ignacio-Espinoza JC, Berlin AM, Kelly L, Weigele PR (2010). Genomic analysis of oceanic cyanobacterial myoviruses compared with T4-like myoviruses from diverse hosts and environments. Environ Microbiol.

[63] Gregory AC, Zayed AA, Conceição-Neto N, Temperton B, Bolduc B, Alberti A (2019). Marine DNA viral macro- and microdiversity from pole to pole. Cell.

[64] Mella-Flores D, Mazard S, Humily F, Partensky F, Mahé F, Bariat L (2011). Is the distribution of prochlorococcus and synechococcus ecotypes in the Mediterranean Sea affected by global warming?. Biogeosciences.

[65] Leconte J, Benites LF, Vannier T, Wincker P, Piganeau G, Jaillon O (2020). Genome resolved biogeography of mamiellales. Genes.

[66] Gallienne CP, Robins DB (2001). Is Oithona the most important copepod in the world’s oceans?. J Plankton Res.

[67] Humes AG (1994). How many copepods?. Hydrobiologia.

[68] Cornils A, Wend-Heckmann B, Held C (2017). Global phylogeography of Oithona similis s.l. (Crustacea, Copepoda, Oithonidae) – a cosmopolitan plankton species or a complex of cryptic lineages?. Mol Phylogenet Evol.

[69] Aarbakke ONS, Bucklin A, Halsband C, Norrbin F (2014). Comparative phylogeography and demographic history of five sibling species of Pseudocalanus (Copepoda: Calanoida) in the North Atlantic Ocean. J Exp Mar Biol Ecol.

[70] Blanc-Mathieu R, Krasovec M, Hebrard M, Yau S, Desgranges E, Martin J (2017). Population genomics of picophytoplankton unveils novel chromosome hypervariability. Sci Adv.

[71] Hirai J, Tsuda A, Goetze E (2015). Extensive genetic diversity and endemism across the global range of the oceanic copepod Pleuromamma abdominalis. Prog Oceanogr.

[72] Kulagin DN, Stupnikova AN, Neretina TV, Mugue NS (2014). Spatial genetic heterogeneity of the cosmopolitan chaetognath Eukrohnia hamata (Möbius, 1875) revealed by mitochondrial DNA. Hydrobiologia.

[73] Stupnikova AN, Molodtsova TN, Mugue NS, Neretina TV (2013). Genetic variability of the Metridia lucens complex (Copepoda) in the Southern Ocean. J Mar Syst.

[74] Sokolov S, Rintoul SR (2009). Circumpolar structure and distribution of the antarctic circumpolar current fronts: 1. Mean circumpolar paths. J Geophys Res Oceans.

[75] Goni G, Kamholz S, Garzoli S, Olson D (1996). Dynamics of the Brazil-Malvinas confluence based on inverted echo sounders and altimetry. J Geophys Res.

[76] Castellani C, Licandro P, Fileman E, Capua ID, Mazzocchi MG (2016). Oithona similis likes it cool: evidence from two long-term time series. J Plankton Res.

[77] Kitzinger K, Marchant HK, Bristow LA, Herbold CW, Padilla CC, Kidane AT, et al. Single cell analyses reveal contrasting life strategies of the two main nitrifiers in the ocean. Nat Commun. 2020;in press. 10.1038/s41467-020-14542-3 PMC7005884 32034151

[78] Baines SB, Twining BS, Brzezinski MA, Krause JW, Vogt S, Assael D (2012). Significant silicon accumulation by marine picocyanobacteria. Nat Geosci.

[79] Ohnemus DC, Rauschenberg S, Krause JW, Brzezinski MA, Collier JL, Geraci-Yee S (2016). Silicon content of individual cells of Synechococcus from the North Atlantic Ocean. Mar Chem.

[80] Karl DM (2014). Microbially mediated transformations of phosphorus in the sea: new views of an old cycle. Annu Rev Mar Sci.

[81] Levitus S, Conkright ME, Reid JL, Najjar RG, Mantyla A (1993). Distribution of nitrate, phosphate and silicate in the world oceans. Prog Oceanogr.

[82] Martiny AC, Lomas MW, Fu W, Boyd PW, Chen YL, Cutter GA (2019). Biogeochemical controls of surface ocean phosphate. Sci Adv.

[83] Tyrrell T (1975). The relative influences of nitrogen and phosphorus on oceanic primary production. Ill Med J.

[84] Hawco NJ, McIlvin MM, Bundy RM, Tagliabue A, Goepfert TJ, Moran DM, et al. Minimal cobalt metabolism in the marine cyanobacterium Prochlorococcus. Proc Natl Acad Sci U S A. 2020;12:15740–7. 10.1073/pnas.2001393117 PMC7354930 32576688

[85] Mooy BASV, Rocap G, Fredricks HF, Evans CT, Devol AH (2006). Sulfolipids dramatically decrease phosphorus demand by picocyanobacteria in oligotrophic marine environments. Proc Natl Acad Sci U S A.

[86] Sjöqvist C, Kremp A, Lindehoff E, Båmstedt U, Egardt J, Gross S (2014). Effects of grazer presence on genetic structure of a phenotypically diverse diatom population. Microb Ecol.

[87] Benedetti F, Vogt M, Elizondo UH, Righetti D, Zimmermann NE, Gruber N (2021). Major restructuring of marine plankton assemblages under global warming. Nat Commun.

[88] Alberti A, Poulain J, Engelen S, et al. Viral to metazoan marine plankton nucleotide sequences from the Tara Oceans expedition. Sci Data. 2017;4:170093. 10.1038/sdata.2017.93. 10.1038/sdata.2017.93 PMC5538240 28763055

[89] Pesant S, Not F, Picheral M, et al. Open science resources for the discovery and analysis of Tara Oceans data. Sci Data. 2015;2:150023. 10.1038/sdata.2015.23. 10.1038/sdata.2015.23 PMC4443879 26029378

[90] Ester M, Kriegel H-P, Sander J, Xu XA. Density-based algorithm for discovering clusters in large spatial databases with noise. 2nd international conference on knowledge discovery and data mining, Portland, OR; 1996. p. 226–231.

[91] Ram A, Jalal S, Jalal A, Manoj K. A density based algorithm for discovering density varied clusters in large spatial databases. Int J Comput Appl. 2010;3:06.

[92] Buchfink B, Xie C, Huson DH (2015). Fast and sensitive protein alignment using DIAMOND. Nat Methods.

[93] Keeling PJ, Burki F, Wilcox HM, Allam B, Allen EE, Amaral-Zettler LA (2014). The Marine Microbial Eukaryote Transcriptome Sequencing Project (MMETSP): illuminating the functional diversity of eukaryotic life in the oceans through transcriptome sequencing. PLoS Biol.

[94] Weir BS, Cockerham CC (1984). Estimating F-statistics for the analysis of population structure. Evolution.

[95] El-Geziry TM, Bryden IG (2010). The circulation pattern in the Mediterranean Sea: issues for modeller consideration. J Oper Oceanogr.

[96] Wu P, Haines K (1996). Modeling the dispersal of Levantine Intermediate Water and its role in Mediterranean deep water formation. J Geophys Res C Oceans.

[97] Laporte F, Charcosset A, Mary-Huard T (2022). Efficient ReML inference in variance component mixed models using a Min-Max algorithm. PLOS Comput Biol.

[98] Lê S, Josse J, Husson F (2008). FactoMineR: an R package for multivariate analysis. J Stat Softw.

[99] van der Maaten L, Hinton G (2008). Visualizing data using t-SNE. J Mach Learn Res.

