# Supplementary material for: Holistic view of the seascape dynamics and environment impact on macro-scale genetic connectivity of marine plankton populations
Source: BMC Ecol Evol. 2023 Sep 1;23:46. doi: 10.1186/s12862-023-02160-8 (PMC10472650; doi:10.1186/s12862-023-02160-8)
Supplement: Supplementary file 2 — Additional file 2: Supplementary Figure S1. Occurrence of species. Species are noted “MVS” for metavariant species. Supplementary Figure S2. Distributions of pairwise-FST by species. Supplementary Figure S3. Lagrangian estimates matrices. Supplementary Figure S4. Lagrangian trajectories for stations of Southern Ocean. Supplementary Figure S5. SNPs clustering with metaVaR. Supplementary Figure S6. Overview of the taxonomic assignment procedure. Supplementary Figure S7. Environmental parameters maps. Supplementary Figure S8. Principal component analysis of the contribution of environmental parameters to the genomic differentiation of plankton species. Supplementary Table S2. Species assigned to Bathycoccus. [file 12862_2023_2160_MOESM2_ESM.docx]

Holistic View of the Seascape Dynamics and Environment Impact on Macro-scale Genetic Connectivity of Marine Plankton Populations

Romuald Laso-Jadart^1,4*^, Michael O’Malley^2^, Adam Sykulski^2^, Christophe Ambroise^3^, Mohammed-Amin Madoui^1,4*^

^1^Génomique Métabolique, Genoscope, Institut François Jacob, CEA, CNRS, Univ Evry, Université Paris-Saclay, Evry, France.

^3^LaMME, CNRS, Univ Evry, Université Paris-Saclay, Evry, France

^6^Research Federation for the study of Global Ocean Systems Ecology and Evolution, FR2022/Tara Oceans GO-SEE, 3 rue Michel-Ange, 75016 Paris, France

^*^Corresponding authors. Emails: rlasojad@genoscope.cns.fr&[amadoui@genoscope.cns.fr](mailto:amadoui@genoscope.cns.fr)

Table of contents

[Supplementary Figure S1: Occurrence of species. Species are noted “MVS” for metavariant species. 3](#_Toc121150447)

[Supplementary Figure S2: Distributions of pairwise-*F_ST_* by species 4](#_Toc121150448)

[Supplementary Figure S3: Lagrangian estimates matrices 5](#_Toc121150449)

[Supplementary Figure S4: Lagrangian trajectories for stations of Southern Ocean. 6](#_Toc121150450)

[Supplementary Figure S5: SNPs clustering with metaVaR 9](#_Toc121150451)

[Supplementary Figure S6: Overview of the taxonomic assignment procedure 10](#_Toc121150452)

[Supplementary Figure S7: Environmental parameters maps 11](#_Toc121150453)

[Supplementary Figure S8: Principal component analysis of the contribution of environmental parameters to the genomic differentiation of plankton species 14](#_Toc121150454)

[Supplementary Table S2: Species assigned to *Bathycoccus* 15](#_Toc121150455)

Supplementary Figure S1: Occurrence of species.

The y axis corresponds the number of species noted “MVS” for Metavariant species.


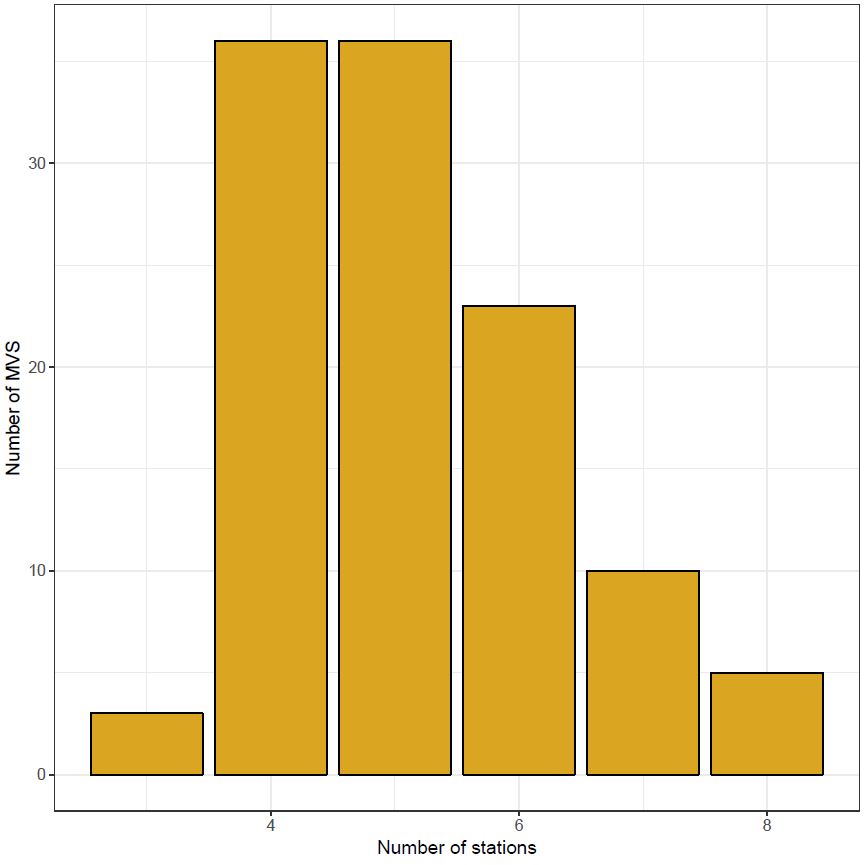


Supplementary Figure S2: Distributions of pairwise-*F_ST_* by species

Each plot corresponds to the distribution of the pairwise-FST for one species. The color of the violin is linked to the taxonomy, and the background color of species’ ID stands for the size fractions; red, blue, green and yellow for 0.8-5µm, 5-20µm, 20-180µm and 180-2000µm respectively.


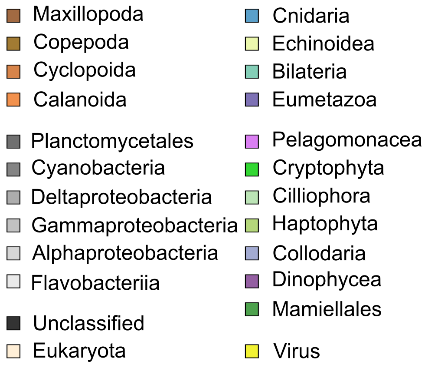


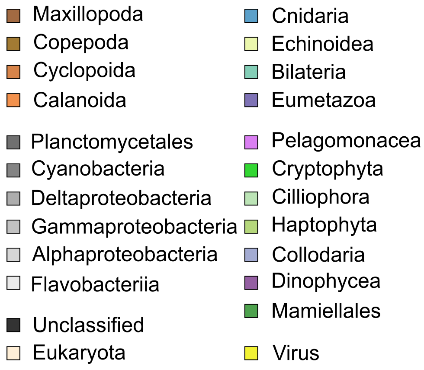


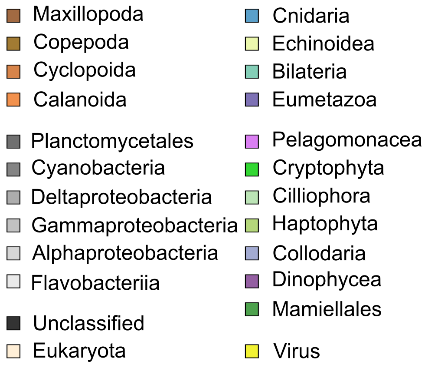


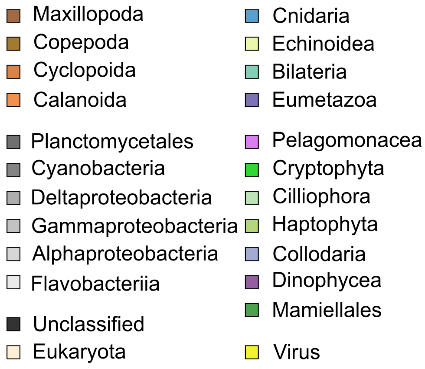


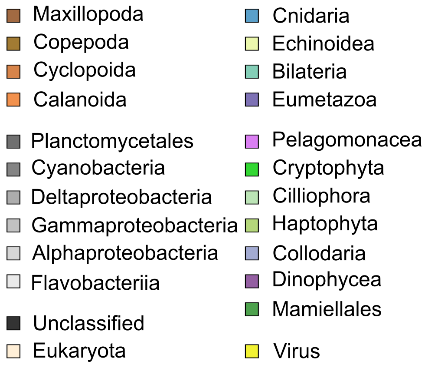


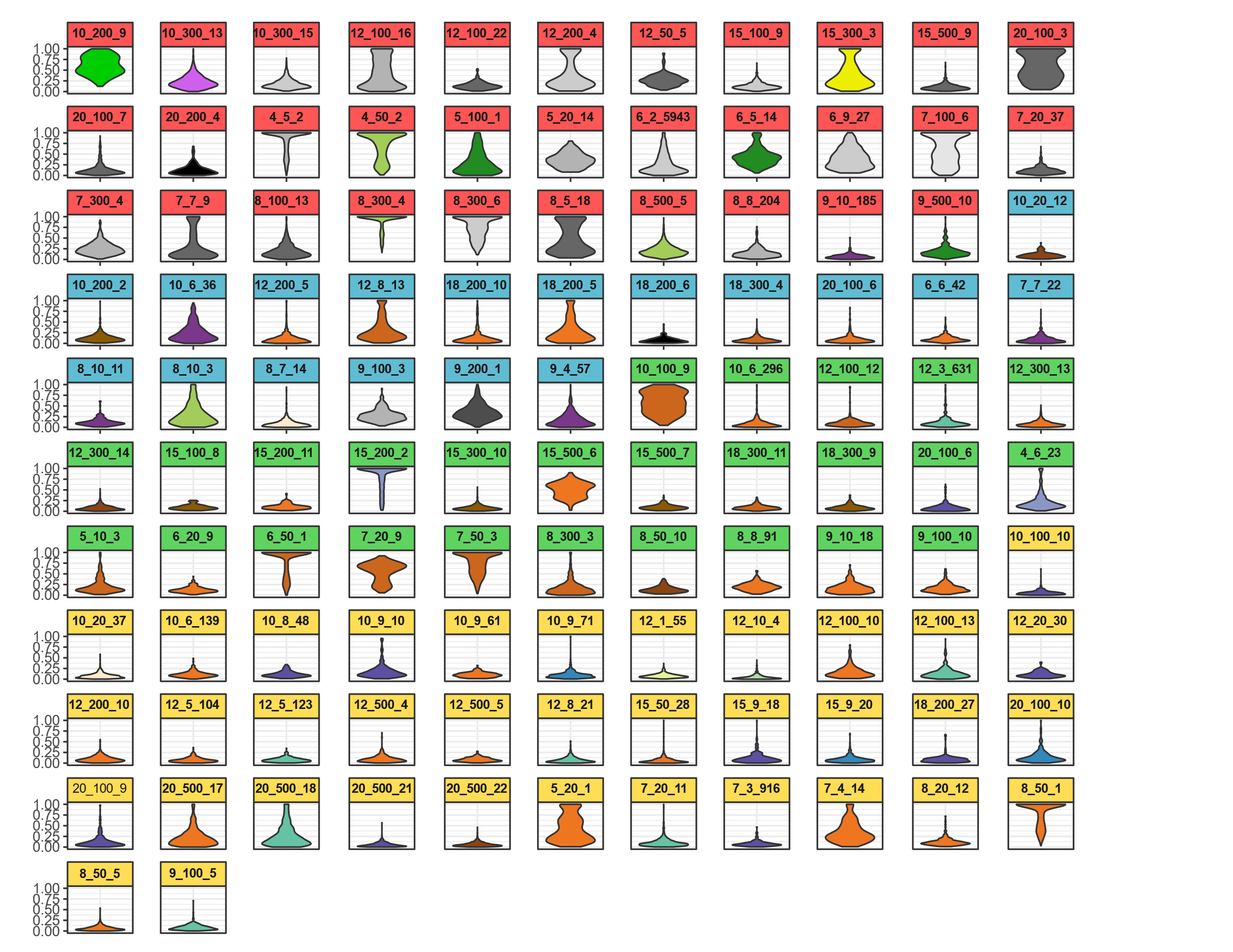


Supplementary Figure S3: Lagrangian estimates matrices

Results of Lagrangian travel time computations. A) Asymmetric times between the 35 stations. Because of the important difference in travel times between Mediterranean Sea stations and the rest, we also present the Lagrangian estimates between B) Mediterranean Sea stations, C) Atlantic and Southern Oceans stations. Based on recorded drifter motion throughout the oceans, we computed Lagrangian travel time estimates between the 35 *Tara* stations, and observed three clear patterns, distinguishing the MED, NAO and SAO/SO. These results showed the relative proximity from TARA_66 to 76 (SAO) and to other NAO stations. We noted a relatively high water masses connectivity from SO stations to TARA_66 and 70 compared to their large geographic distance. The station TARA_145 was isolated to the rest of NAO stations. The Mediterranean stations TARA_7, 9 and 11 were also isolated to the other Mediterranean stations


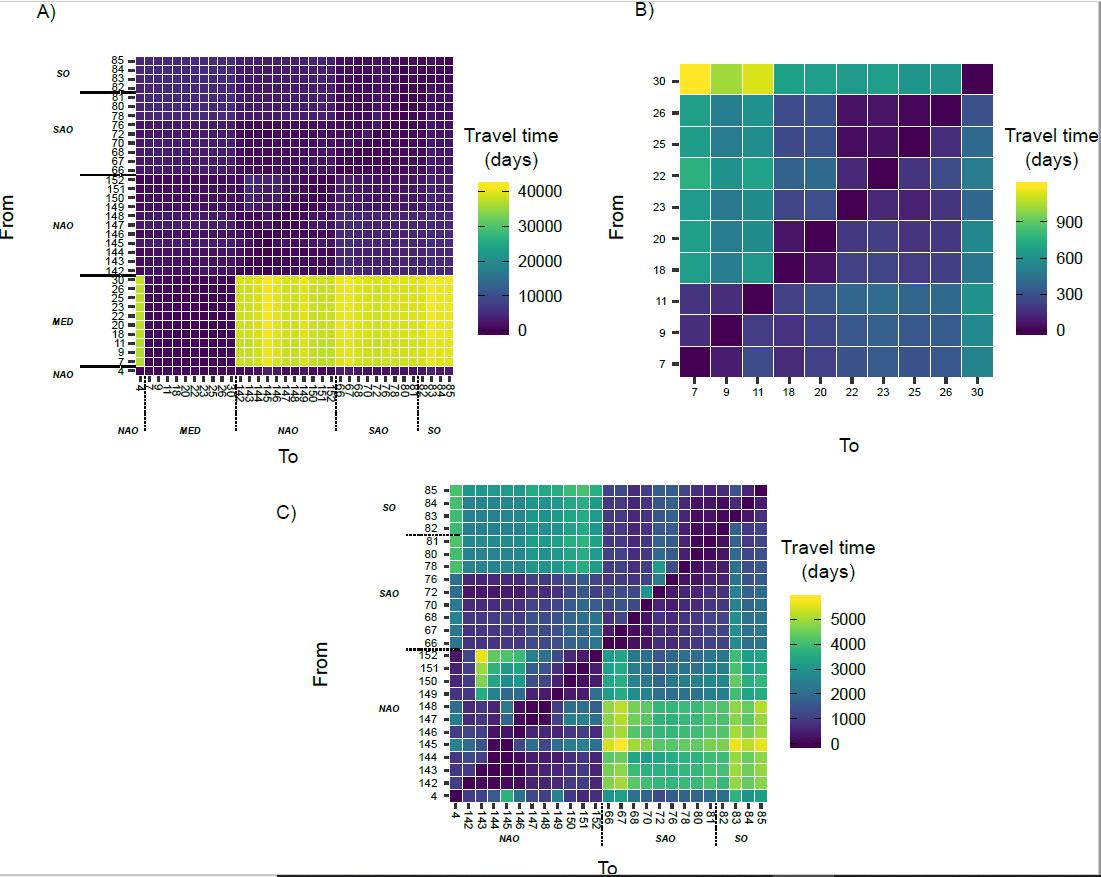


Supplementary Figure S4: Lagrangian trajectories for stations of Southern Ocean.

For each pair of stations; the two upper plots are the drifters trajectories with the fastest and the slowest tracks in blue and red respectively and the lower plots picture the distribution of Lagrangian travel times estimated after bootstrap for the corresponding trajectory.


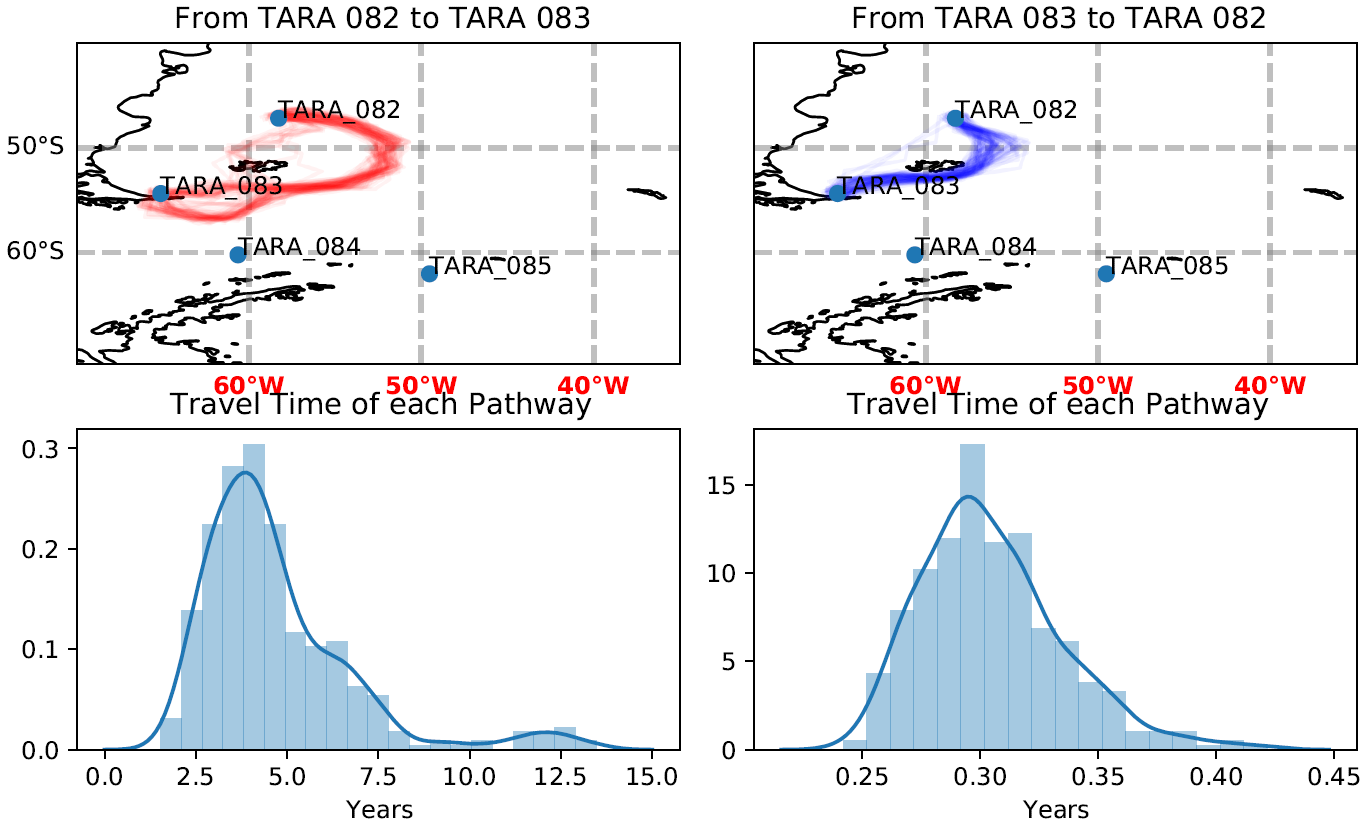

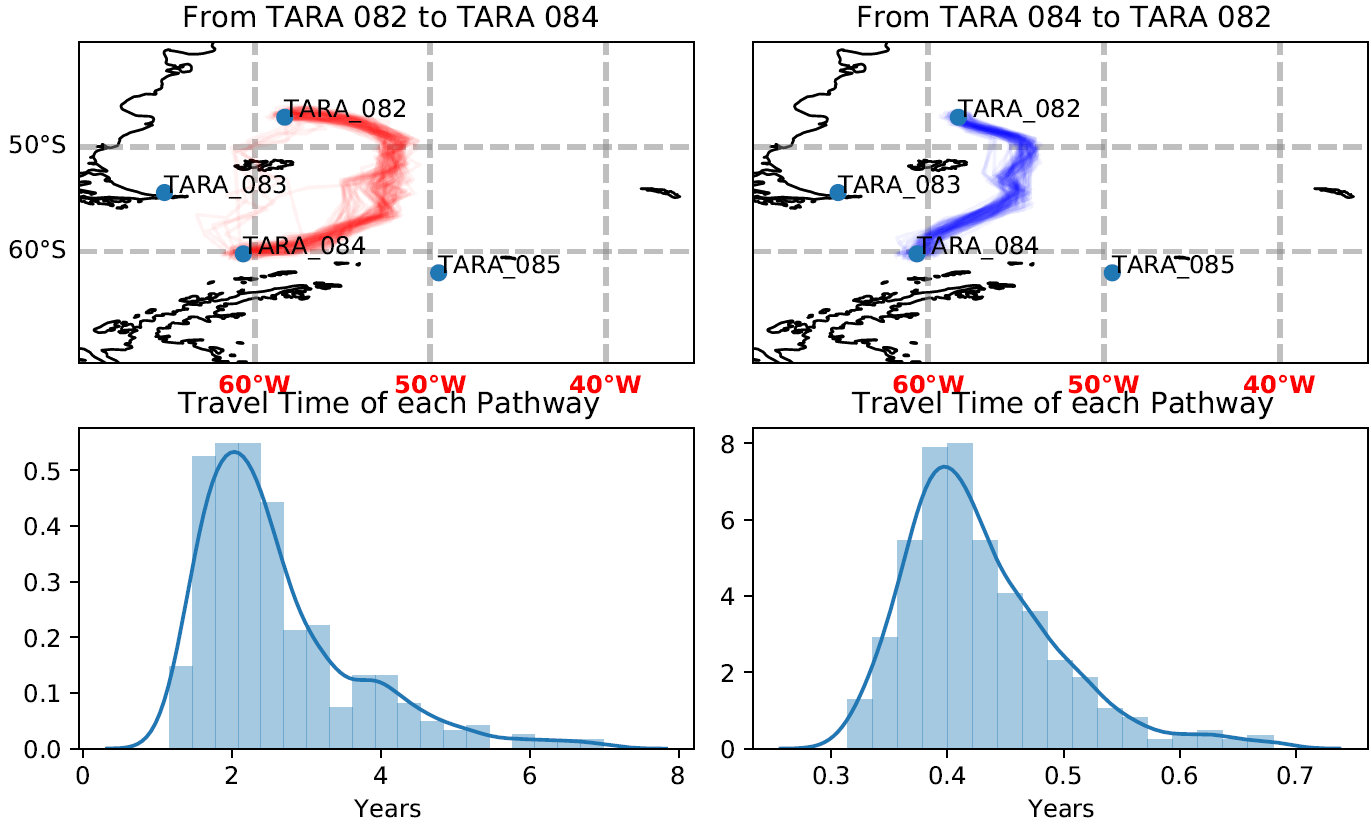


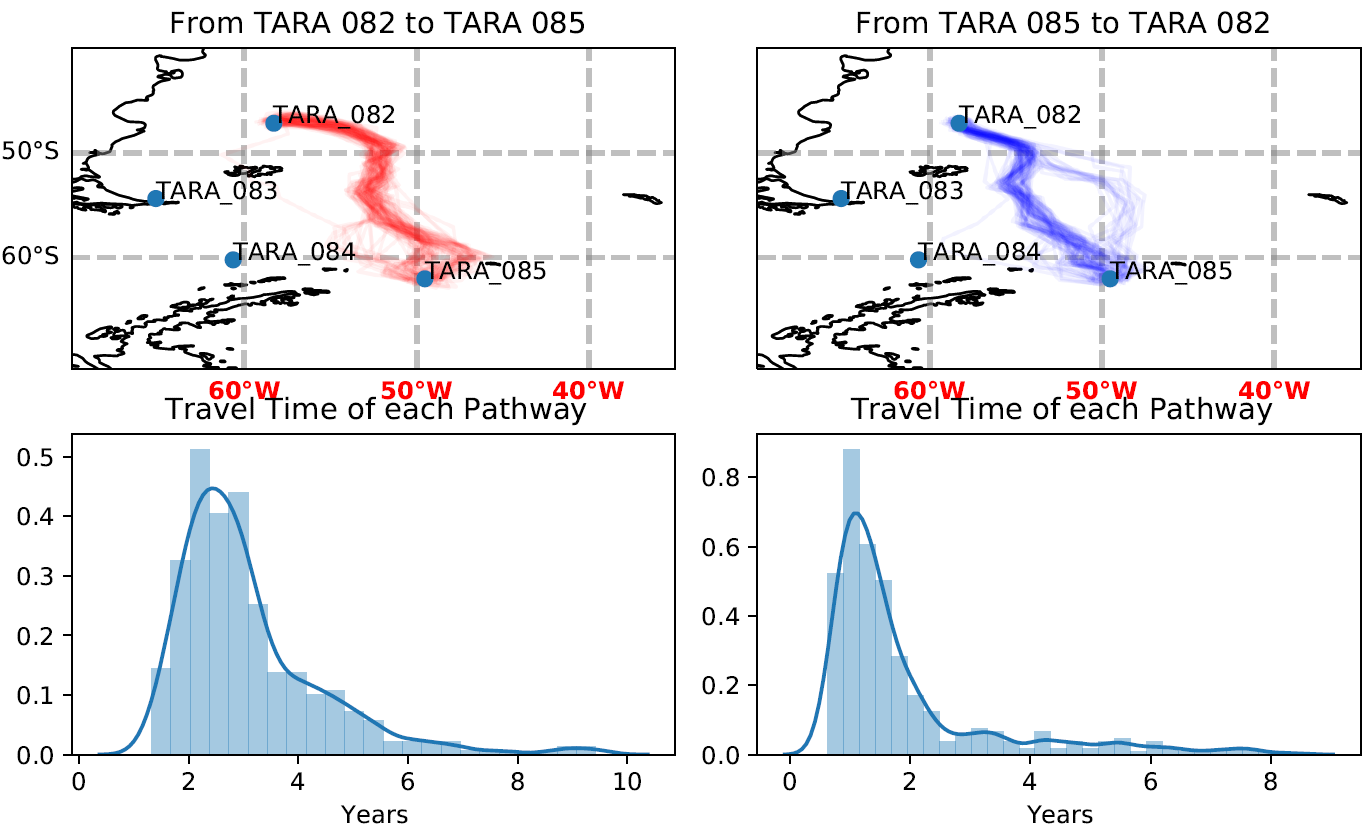


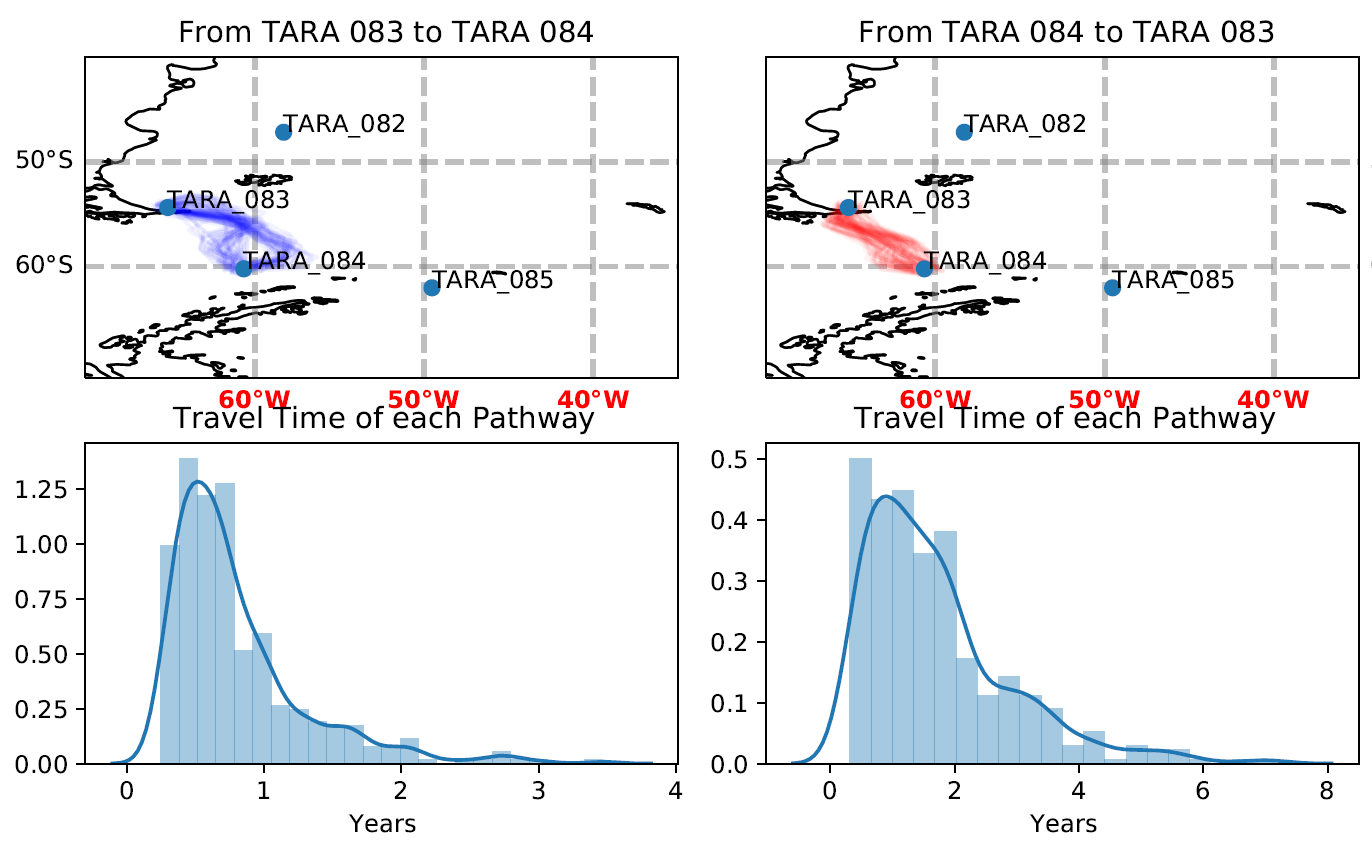


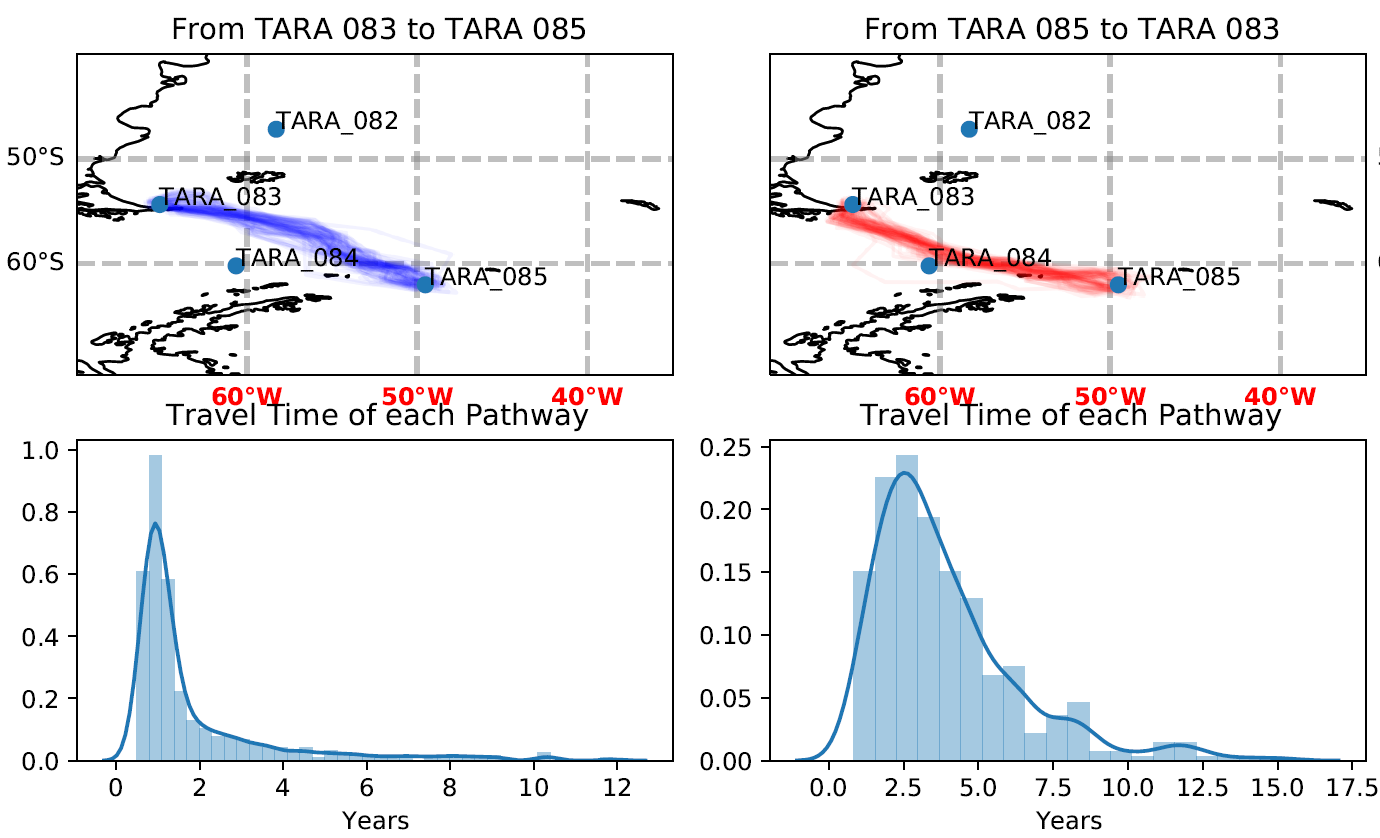


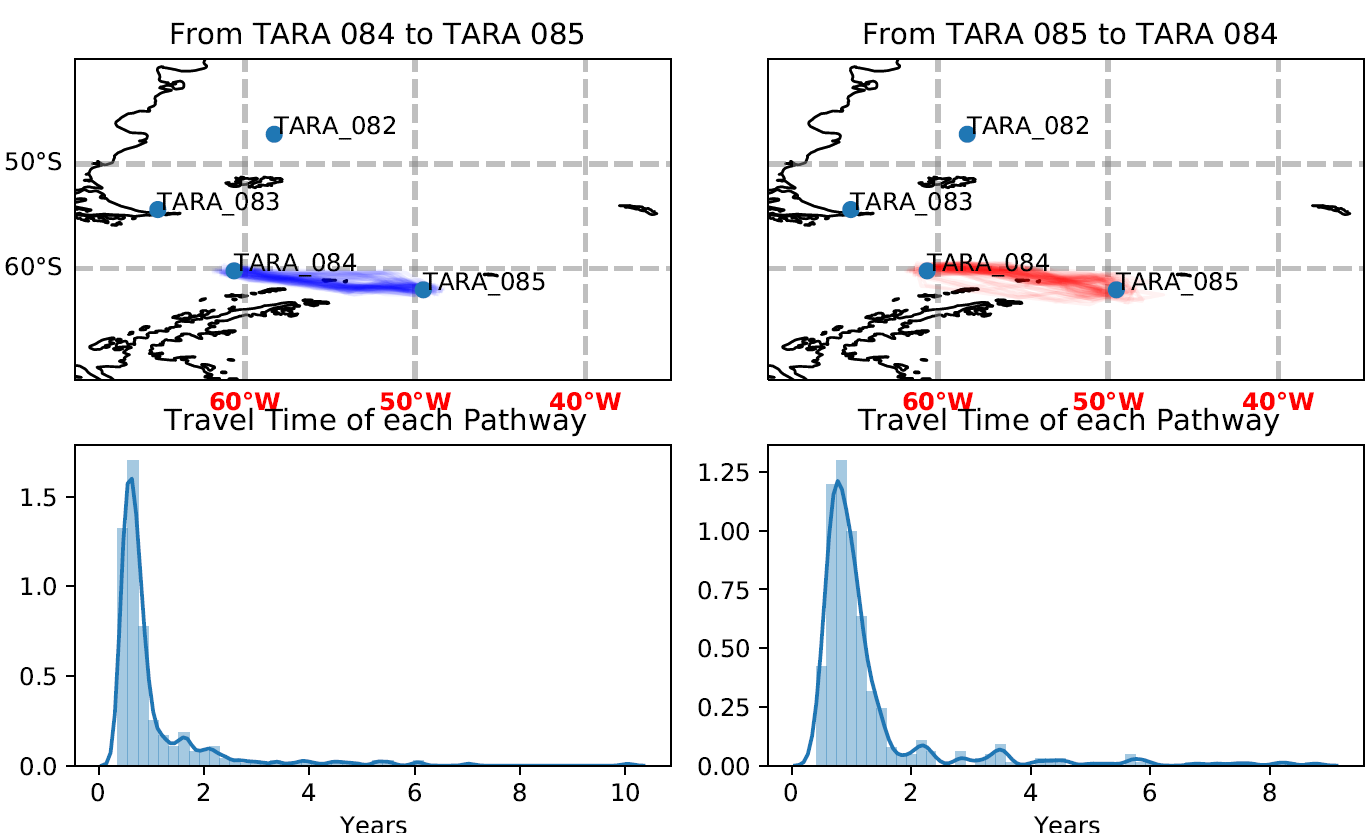


Supplementary Figure S5: SNPs clustering with metaVaR

Number of cluster of SNPs (called MVC) found for each dataset, and for each couple of dbscsan parameters ε and minimum points (MinPts). In blank, no cluster was found for the corresponding parameters.


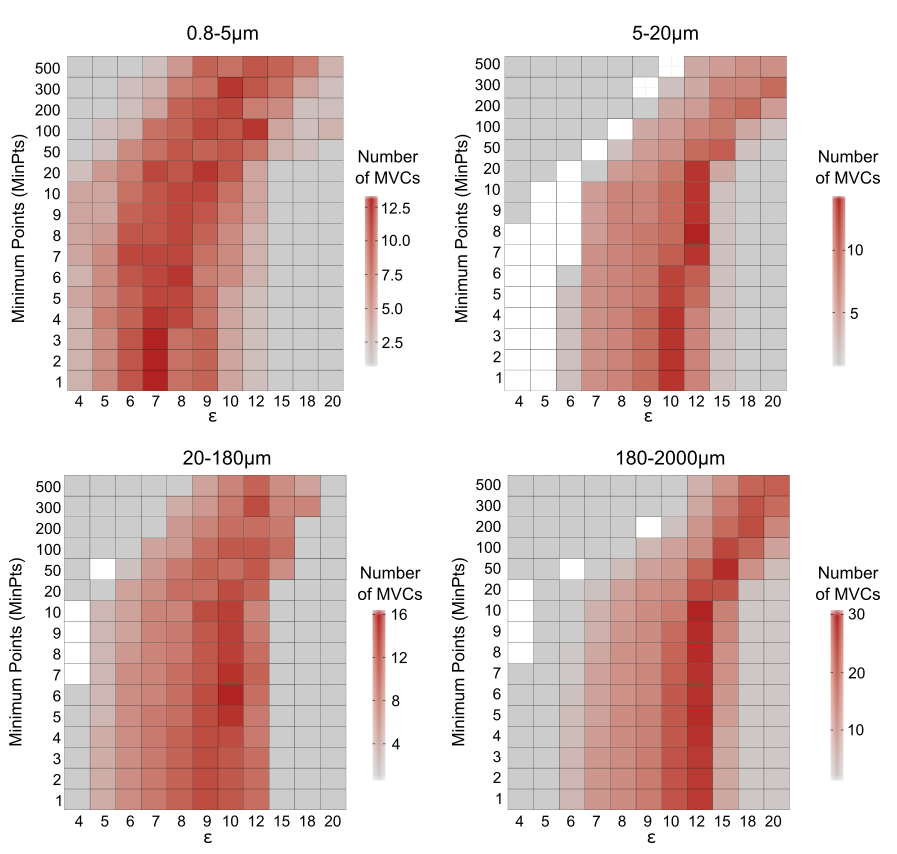


Supplementary Figure S6: Overview of the taxonomic assignment procedure

Pipeline describing how each species (called MVS) was assigned to a taxonomic group.


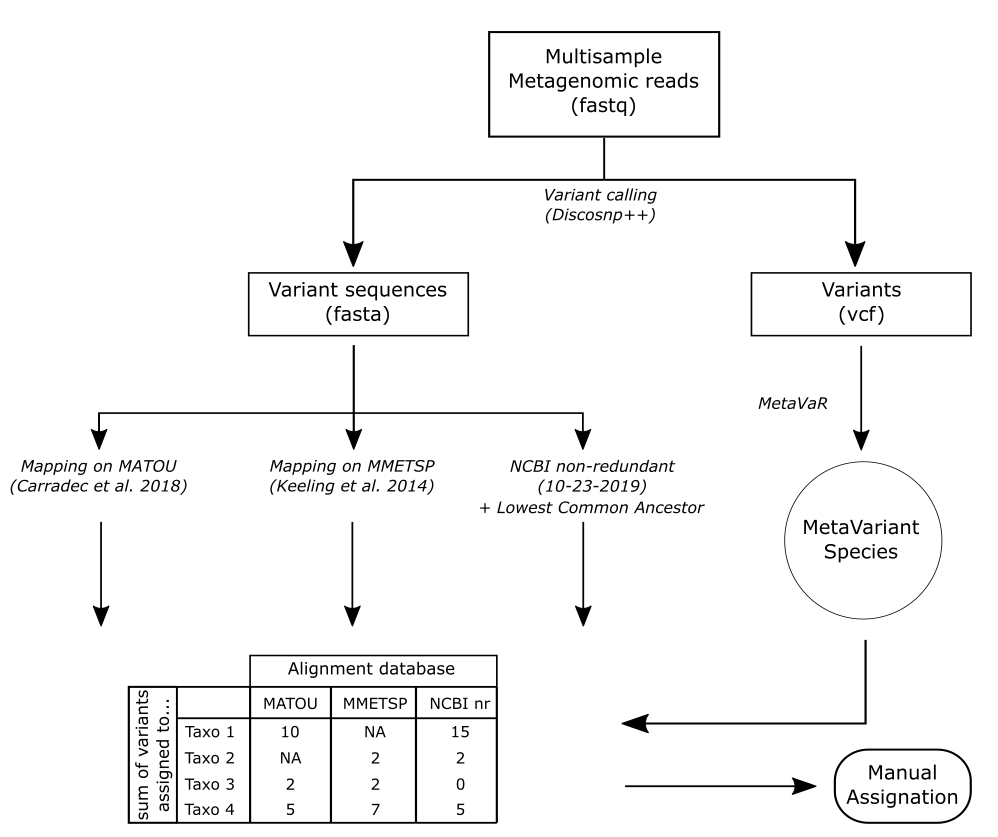


Supplementary Figure S7: Environmental parameters maps

Each dot corresponds to a *Tara* station.


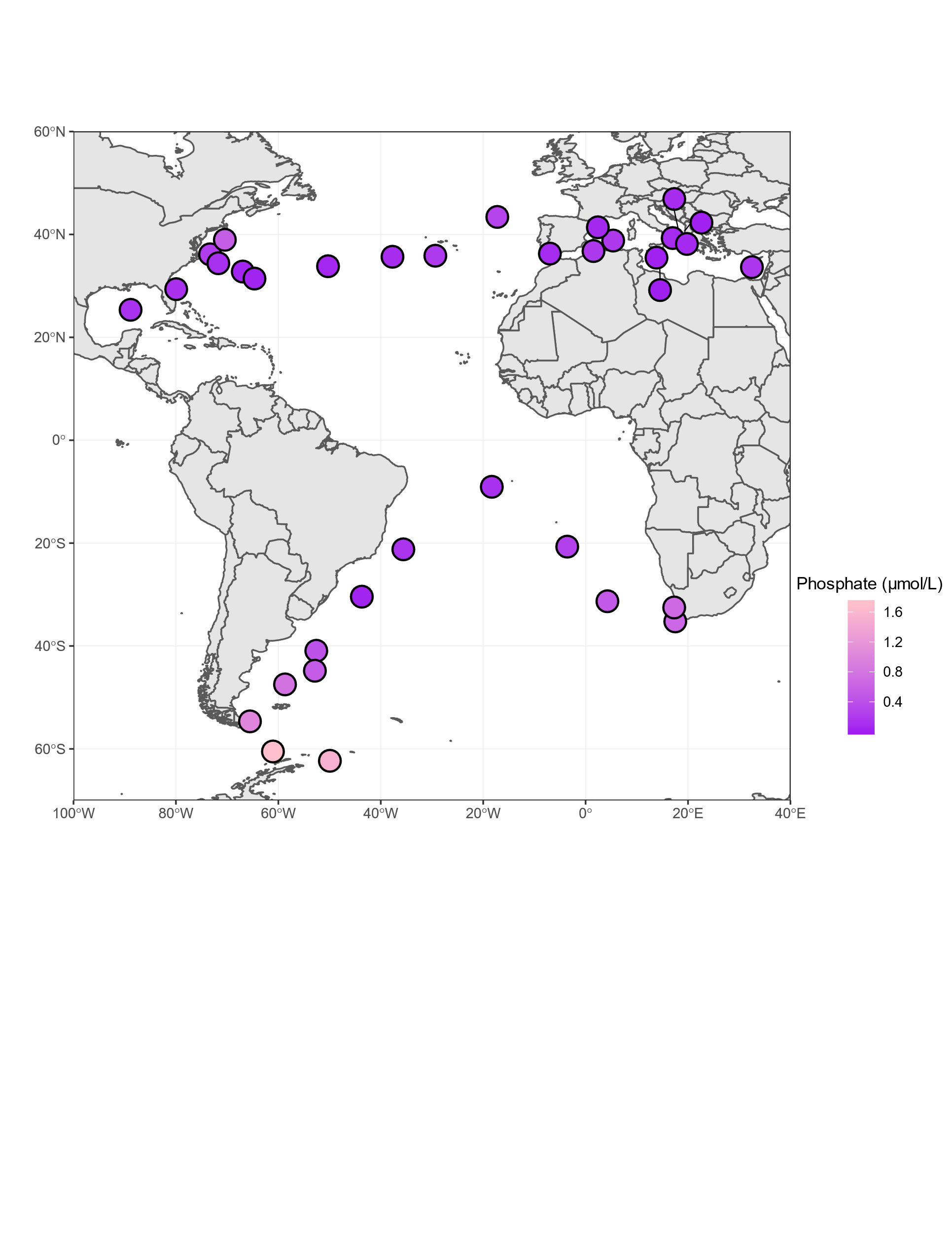


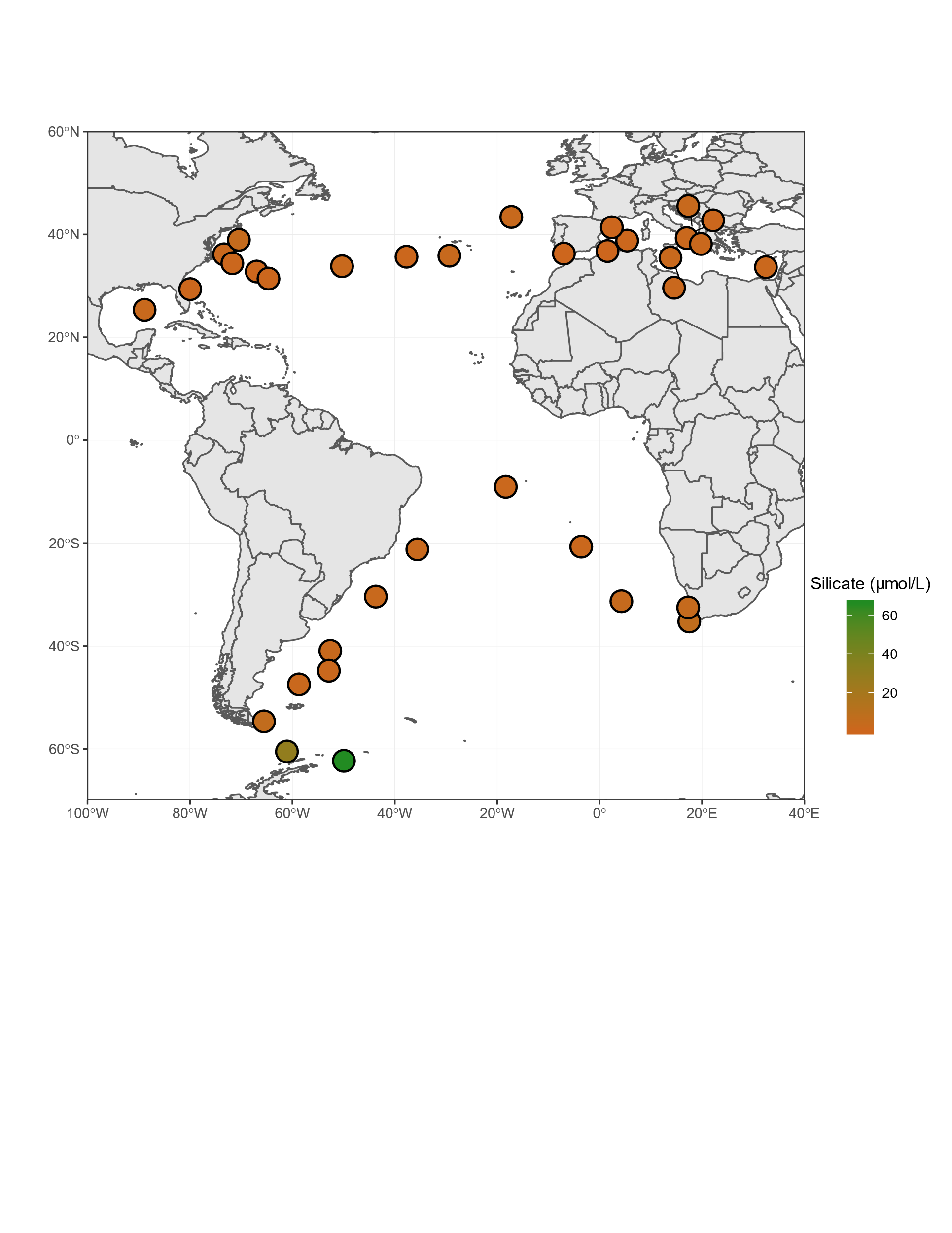

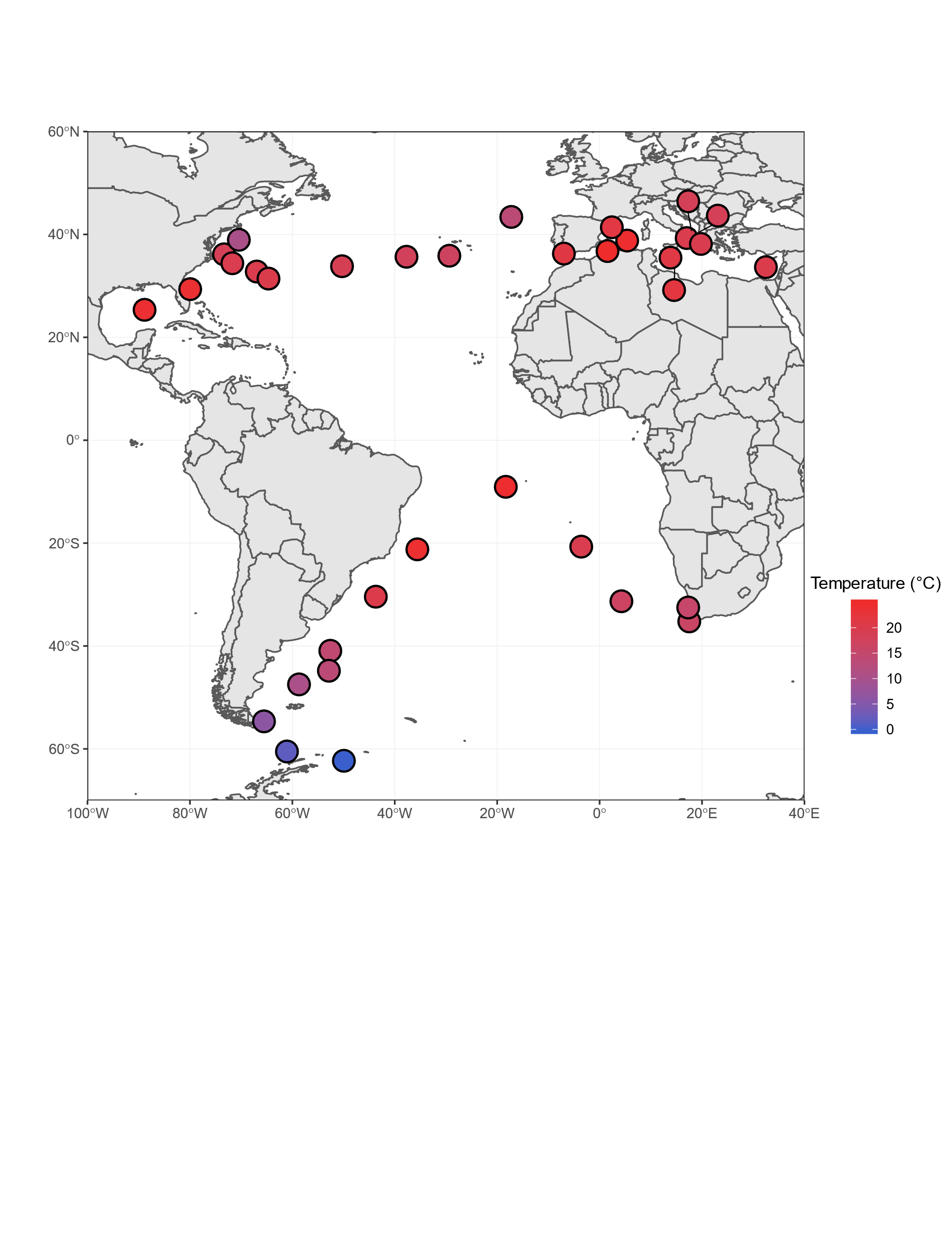


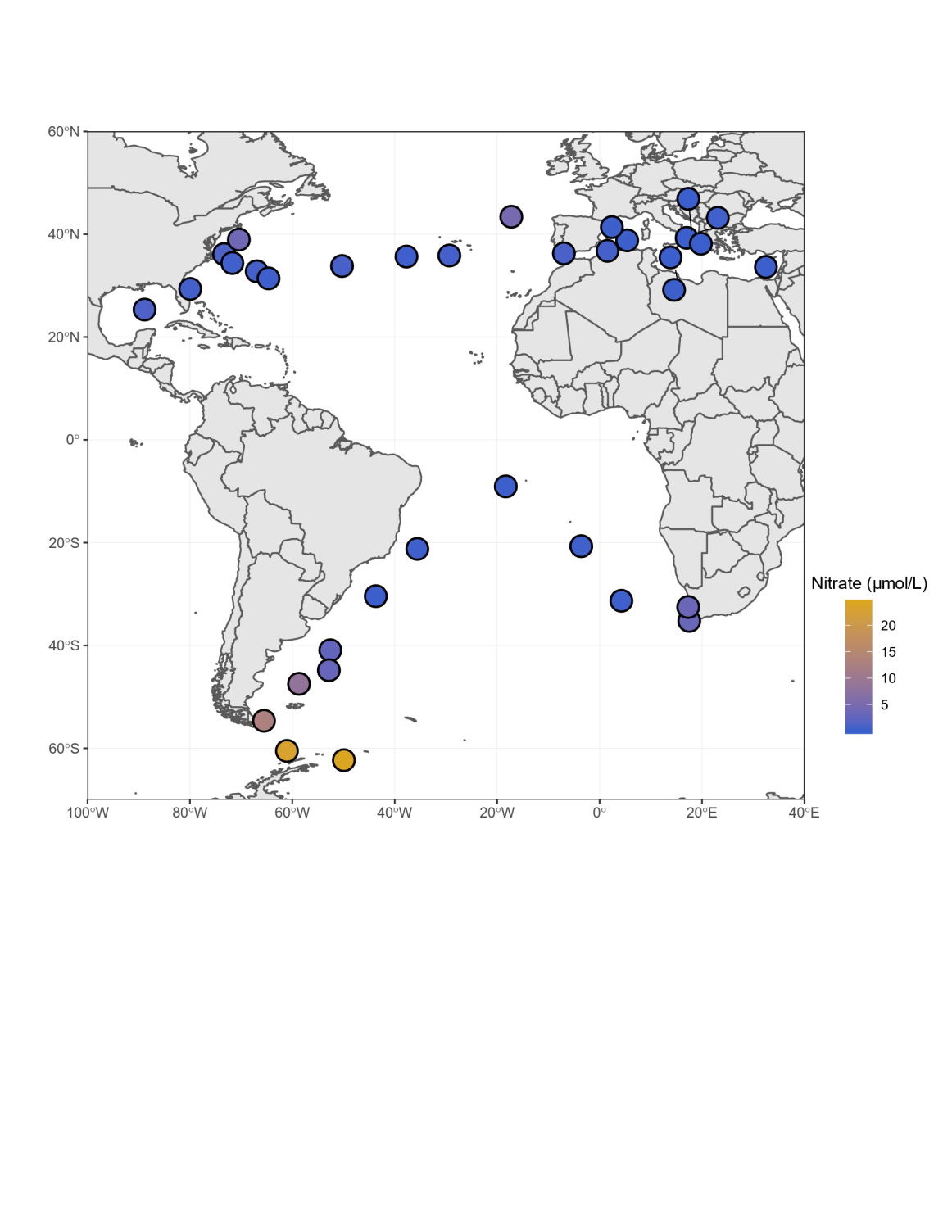


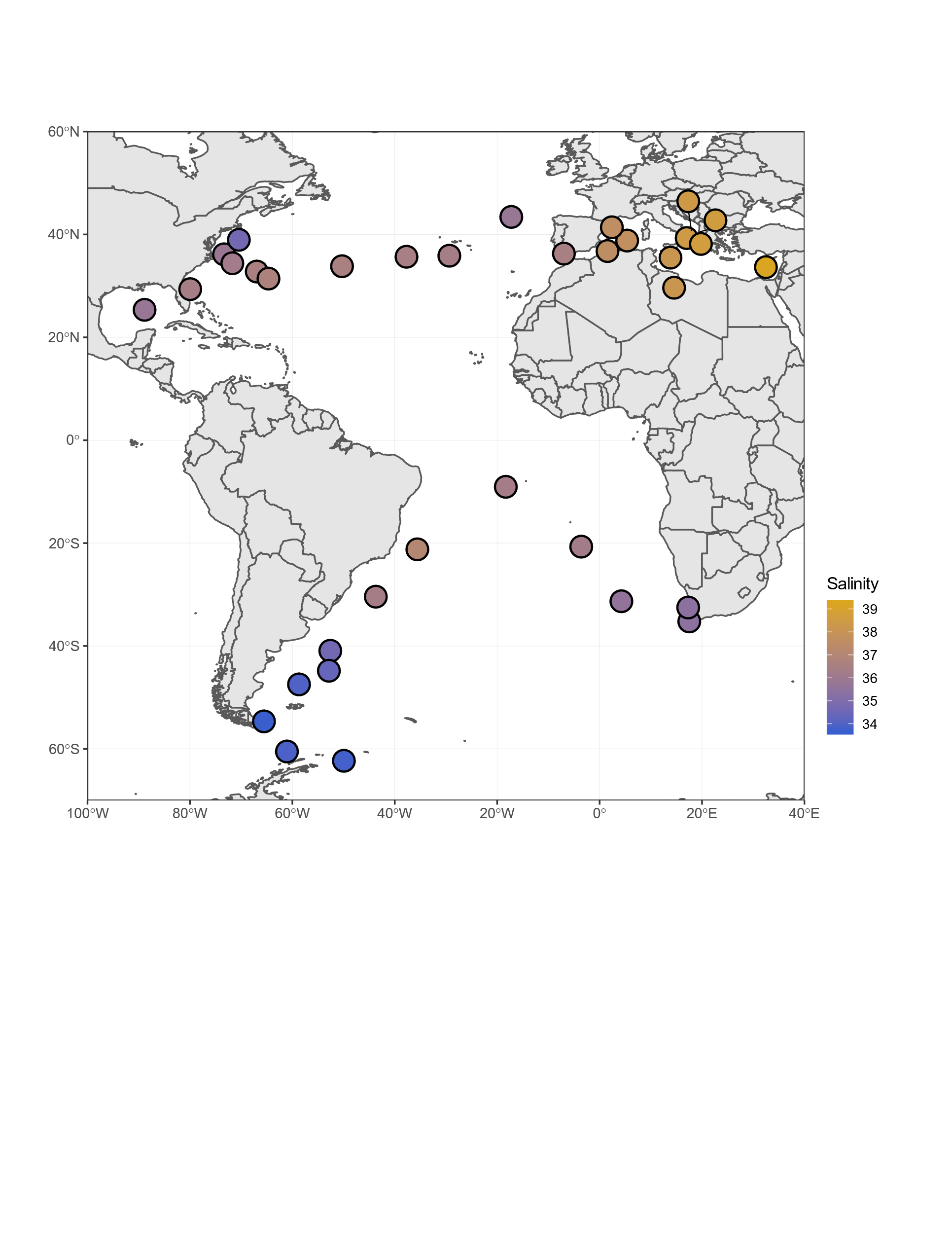


Supplementary Figure S8: Principal component analysis of the contribution of environmental parameters to the genomic differentiation of plankton species


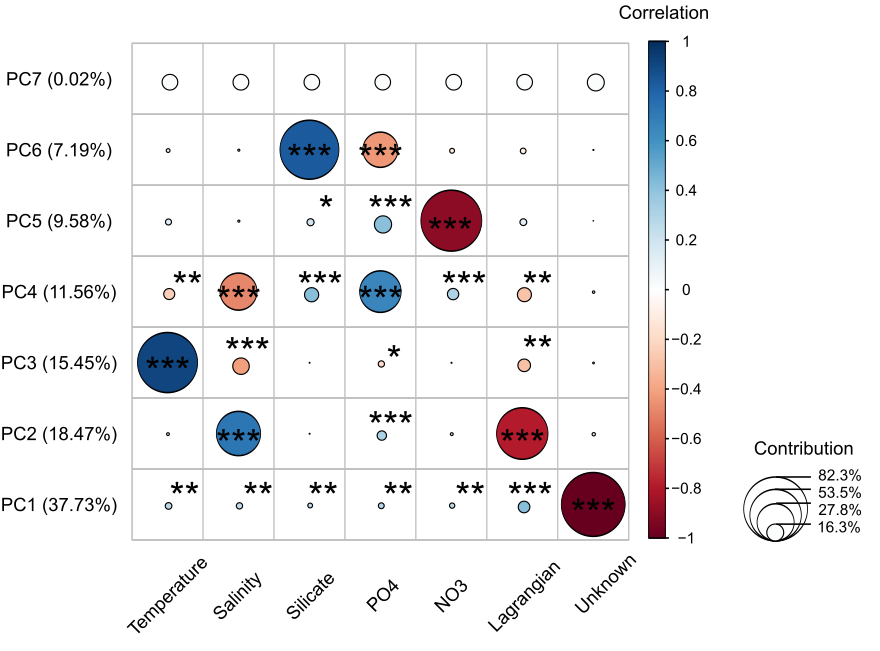


Supplementary Table S2: Species assigned to *Bathycoccus*

The columns *"Bathycoccus"* species reflects the occurrences of the two MVSs identified as potential *Bathycoccus* in our dataset. The columns “*Bathycoccus* strains” are the percentage of metagenomic reads from each *Tara* stations matching the two reference genomes (data extracted from Leconte et al. 2020). Species 6_5_14 and 9_500_10 are present where *Bathycoccusprasinos* RCC1105 and *Bathycoccus* TOSAG39.1 are the most abundant, respectively.

|  | *"Bathycoccus"* species | | *Bathycoccus* strains | |
| --- | --- | --- | --- | --- |
| *Tara* stations | 6_5_14 | 9_500_10 | *Bathycoccus prasinos RCC1105* | *Bathycoccus TOSAG39.1* |
| TARA_66 | Yes | No | 0.7600 | 0.1264 |
| TARA_67 | Yes | No | 0.9098 | 0.0156 |
| TARA_80 | Yes | Yes | 0.9215 | 0.3204 |
| TARA_81 | Yes | No | 1.3416 | 0.0202 |
| TARA_142 | No | No | 0.0005 | 0.0345 |
| TARA_145 | Yes | No | 1.3493 | 0.1263 |
| TARA_146 | No | Yes | 0.1010 | 1.8254 |
| TARA_147 | No | Yes | 0.0906 | 0.8468 |
| TARA_150 | No | Yes | 0.3085 | 0.2685 |
| TARA_152 | Yes | No | 0.4797 | 0.0329 |
